# Supplementary material for: Neurodegenerative VPS41 variants inhibit HOPS function and mTORC1‐dependent TFEB/TFE3 regulation
Source: EMBO Mol Med. 2021 Apr 14;13(5):e13258. doi: 10.15252/emmm.202013258 (PMC8103106; doi:10.15252/emmm.202013258)
Supplement: Supplementary file 1 — Appendix [file EMMM-13-e13258-s003.pdf]

Neurodegenerative *VPS41* variants inhibit HOPS function and mTORC1-dependent TFEB/TFE3 regulation

Reini E.N. van der Welle, Rebekah Jobling, Christian Burns, Paolo Sanza, Corlinda ten Brink, Alfonso Fasano, Lan Chen, Fried J. Zwartkruis, Susan Zwakenberg, Edward F. Griffin, Jan A. van der Beek, Tineke Veenendaal, Nalan Liv, Conny M.A. van Ravenswaaij-Arts, Henny H. Lemmink, Rolph Pfundt, Susan Blaser, Carolina Sepulveda, Andres M. Lozano, Grace Yoon, Teresa Santiago-Sim, Cedric S. Asensio, Guy A. Caldwell, Kim A. Caldwell, David Chitayat, Judith Klumperman

Appendix

Appendix Figure S1

Appendix Figure S2

Appendix Figure S3

Appendix Figure S4

Appendix Figure S5

Appendix Figure S6

Appendix Figure S7

Appendix Figure S8

Appendix Figure S9

Appendix Figure S10

Appendix Figure S11

Appendix Supplementary methods

Appendix Table S1

Appendix Table S2

Appendix Table S3

Appendix Figure S1

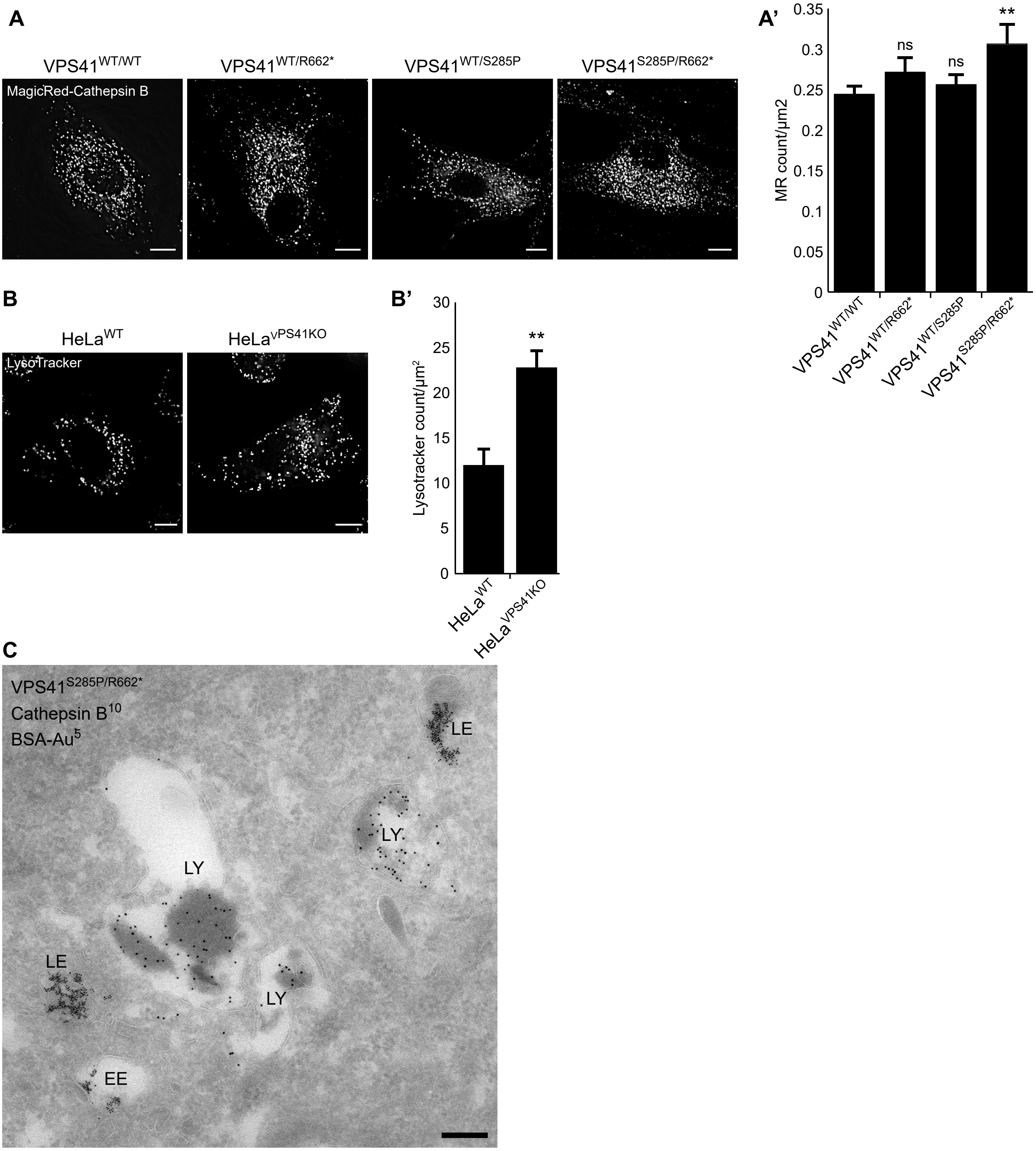

Appendix Figure S1. Mutations in *VPS41* or *VPS41*^{KO} result in increased numbers of enzymatically active, acidified lysosomes.

A. *VPS41*$^{WT/WT}$, *VPS41*$^{WT/S285P}$, *VPS41*$^{WT/R662*}$ and *VPS41*$^{S285P/R662*}$ fibroblasts incubated with MagicRed-Cathepsin B and imaged by fluorescence microscopy to visualize enzymatically active compartments. *VPS41*$^{S285P/R662*}$ fibroblasts show significantly more compartments with active Cathepsin B (quantified in A′) >10 Cells per condition were quantified (n=3). Bars, 10μm.

B. HeLa^{WT} and HeLa^{VPS41KO} cells incubated with LysoTracker Red to assess the prevalence of acidified compartments in the absence of VPS41. Hela^{VPS41KO} cells show a 2-fold increase in number of acidified compartments (quantified in B′) >18 Cells per condition were quantified (n=3). Bars, 10μm.

C. Ultrathin cryosections of *VPS41*$^{S285P/R662*}$ fibroblasts incubated for 2 hours with BSA conjugated to 5 nm gold (BSA-Au^5) and immuno-gold labeled for Cathepsin B (10 nm gold particles). BSA-Au^5 is seen in early and late endosomes that are devoid of Cathepsin B. By contrast, lysosomes are negative for BSA-Au^5 and positive for Cathepsin B. These data indicate that enzymatically active lysosomes are poorly reached by endocytic marker. EE = Early endosome, LE = late endosome, LY = lysosome. Bars, 200nm.

Data information: Data are represented as mean ± SEM. $^{**}P < 0.01$. One-way ANOVA with Bonferroni correction (A′) or Unpaired *t*-test (B′). Exact p-values are reported in Appendix Table S3.

Appendix Figure S2

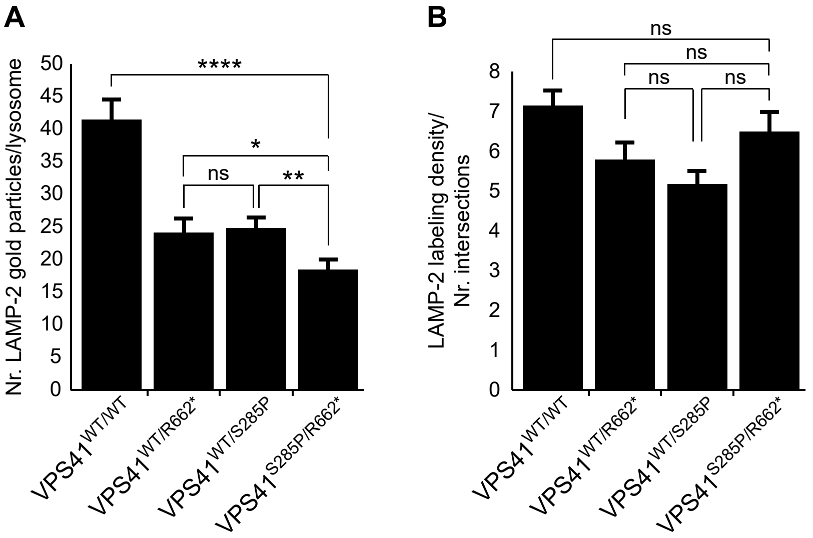

Appendix Figure S2. Small lysosomes of patient fibroblasts contain normal LAMP-2 levels.

A. To determine if lysosomes in patient derived fibroblasts contain LAMP-2, $\mathit{VPS41}^{WT/WT}$, $\mathit{VPS41}^{WT/S285P}$, $\mathit{VPS41}^{WT/R662*}$ and $\mathit{VPS41}^{S285P/R662*}$ fibroblasts were prepared for immuno-EM and labeled for LAMP-2. Numbers represent the amount of LAMP-2 representing gold particles per lysosome. >46 Lysosomes per condition were quantified.

B. Lysosomes in $\mathit{VPS41}^{S285P/R662*}$ fibroblasts are smaller than in control cells. When corrected for lysosome size, measured by number of intersections of a grid (200nm grid size) with the limiting membrane of a specific lysosome, LAMP-2 labeling densities were similar between $\mathit{VPS41}^{WT/WT}$, $\mathit{VPS41}^{WT/S285P}$, $\mathit{VPS41}^{WT/R662*}$ or $\mathit{VPS41}^{S285P/R662*}$ fibroblasts. Hence, patient lysosomes are smaller, but contain similar LAMP-2 concentrations as control cells. >46 Lysosomes per condition were quantified.

Data information: Data are represented as mean ± SEM. $*P < 0.05$, $**P < 0.01$, $****P < 10^{-5}$. One-way ANOVA with Tukey’s correction. Exact p-values are reported in Appendix Table S3.

Appendix Figure S3

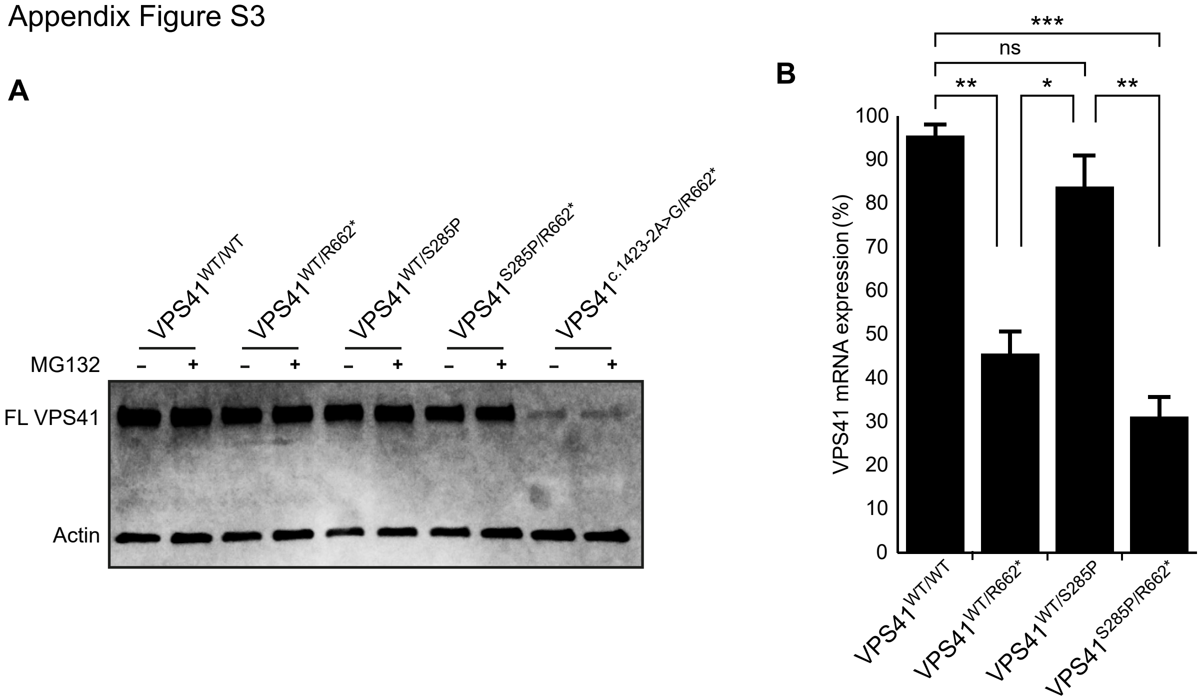

Appendix Figure S3. The *VPS41*^{R662*} variant is not translated in patient and maternal fibroblasts.

A. Longer exposure of western blot also shown in Fig 3A. Analysis of VPS41 protein levels in *VPS41*$^{WT/WT}$, *VPS41*$^{WT/S285P}$, *VPS41*$^{WT/R662*}$, *VPS41*$^{S285P/R662*}$ and *VPS41*$^{1432\text{-}2A>G/R662*}$ fibroblasts with and without MG132 proteasomal inhibition. In none of the conditions the R662* mutant is visible (n=2).

B. RT-PCR analysis of *VPS41* mRNA levels in *VPS41*$^{WT/WT}$, *VPS41*$^{WT/S285P}$, *VPS41*$^{WT/R662*}$ and *VPS41*$^{S285P/R662*}$ fibroblasts. Both maternal and patient fibroblasts bearing the *R662** mutant show a significant decrease in *VPS41* mRNA levels, indicating that the premature stop codon in *R662** leads to premature mRNA degradation (n=3).

Data information: Data are represented as mean ± SEM. $*P < 0.05$, $**P < 0.01$, $***P < 10^{-4}$. One-way ANOVA with Tukey's correction. Exact p-values are reported in Appendix Table S3.

Appendix Figure S4

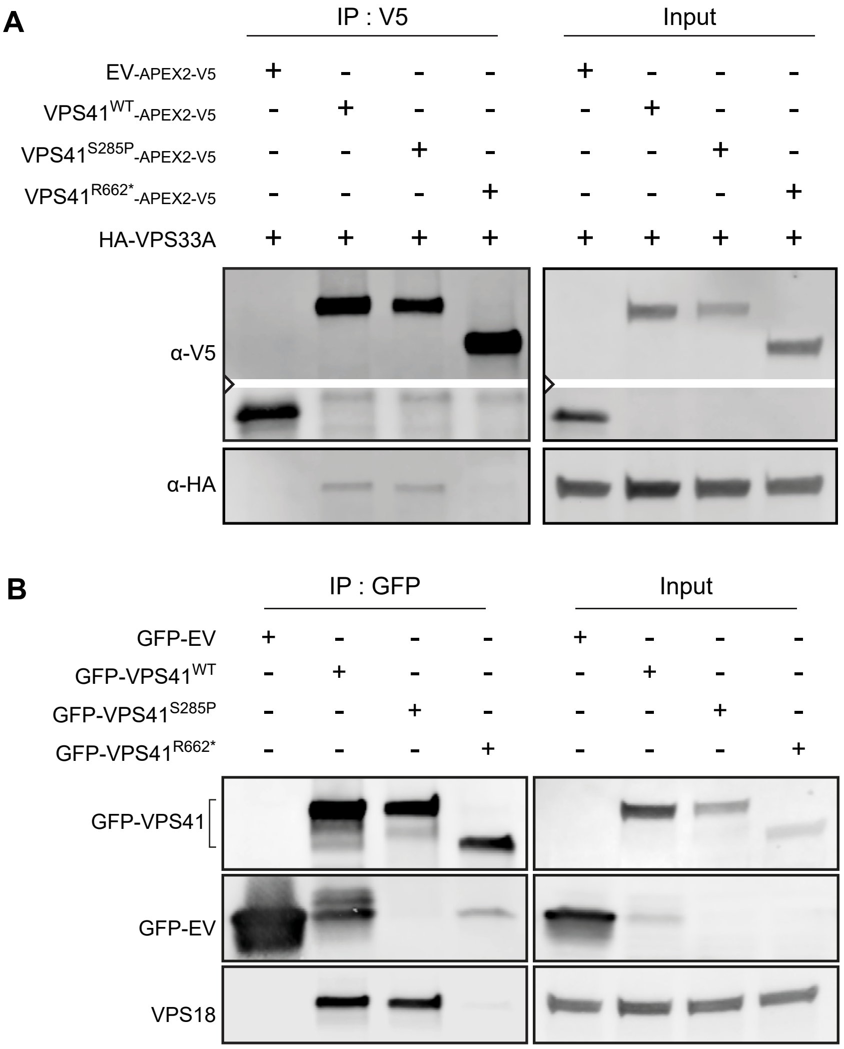

Appendix Figure S4. VPS41^{R662*} does not interact with other HOPS subunits.

A. Immunoprecipitation (IP) on HeLa^{WT} cells co-expressing EV-APEX2-V5, VPS41^{WT}-APEX2-V5, VPS41^{S285P}-APEX2-V5 or VPS41^{R662*}-APEX2-V5 and HA-VPS33A. Western blot analysis shows that, in contrast to VPS41^{WT} and VPS41^{S285P}, the VPS41^{R662*} variant does not interact with VPS33A (n=3).

B. IP on HeLa^{VPS41KO} cells expressing GFP-EV, GFP-VPS41^{WT}, GFP-VPS41^{S285P} or GFP-VPS41^{R662*}. Western blot analysis shows a strong interaction between VPS41^{WT} and VPS41^{S285P} with endogenous VPS18. By contrast, there is no interaction between VPS41^{R662*} and VPS18 (n=2).

Appendix Figure S5

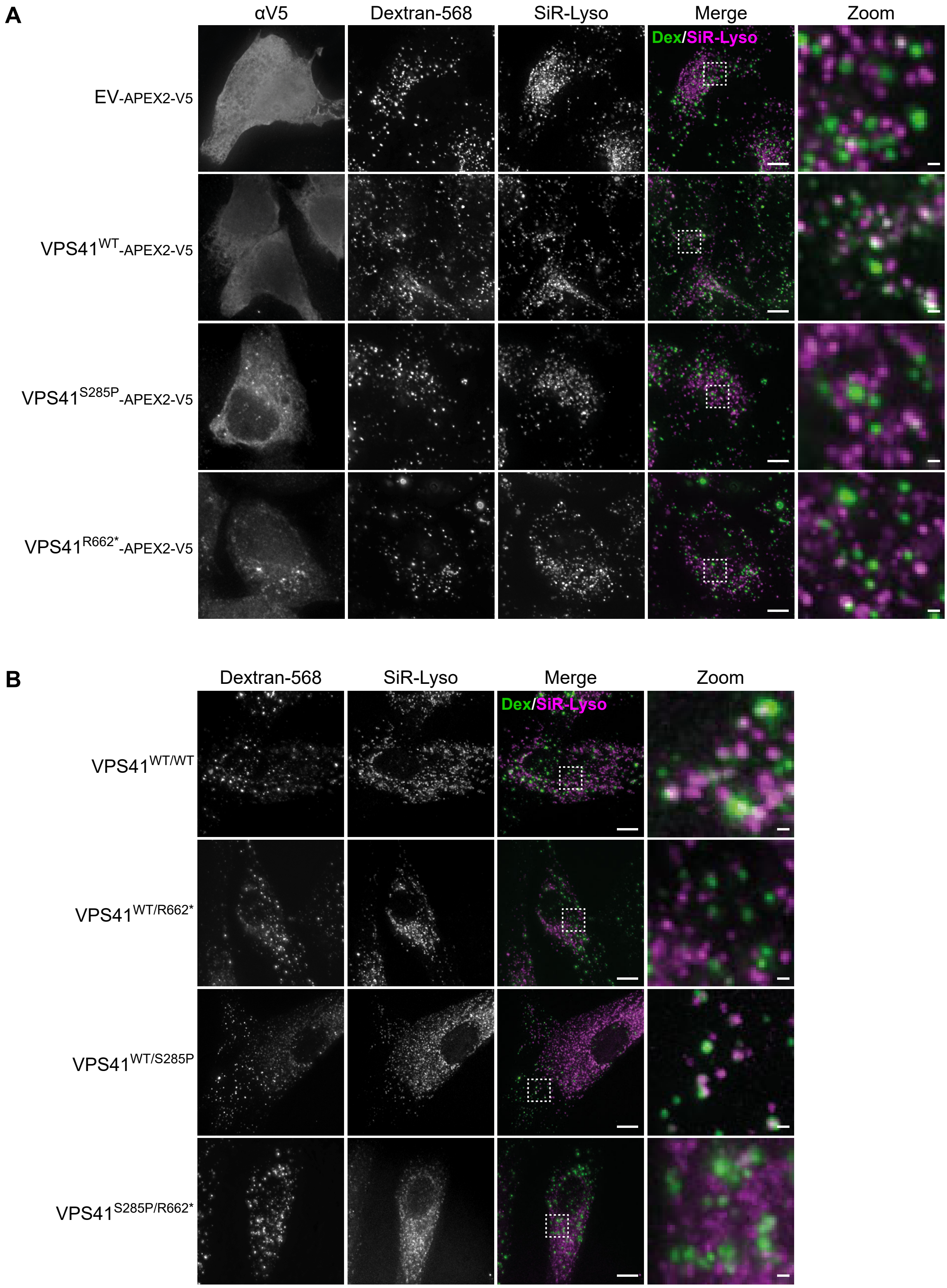

Appendix Figure S5. Expression of VPS41 variants do not restore late endosome – lysosome fusion.

A. Rescue experiments. Transfection of HeLa^{VPS41KO} cells with VPS41^{WT}-APEX2-V5 show a significant increase in co-localization between Dextran-568 and SiR-Lysosome Cathepsin D (SiR-Lyso), indicating rescue of the endocytosis phenotype observed in *VPS41*^{KO} cells. Expression of the *VPS41* variants does not improve colocalization, indicating that these variants fail to rescue HOPS complex functionality (quantified in Fig 3E). Bars, 10μm; zoom, 1μm.

B. Endocytosis phenotype in patient and maternal fibroblasts. *VPS41*$^{WT/WT}$, *VPS41*$^{WT/S285P}$, *VPS41*$^{WT/R662*}$ and *VPS41*$^{S285P/R662*}$ primary fibroblasts were incubated with Dextran-568 and SiR-Lysosome Cathepsin D (SiR-Lyso) for 2 and 3 hours, respectively. Co-localization representing delivery of Dextran to enzymatically active lysosomes was decreased in *VPS41*$^{WT/R662*}$ and *VPS41*$^{S285P/R662*}$ cells, indicating reduced fusion efficiency between late endosomes and lysosomes (quantified in Fig 3F). Bars, 10μm; zoom, 1μm.

Appendix Figure S6

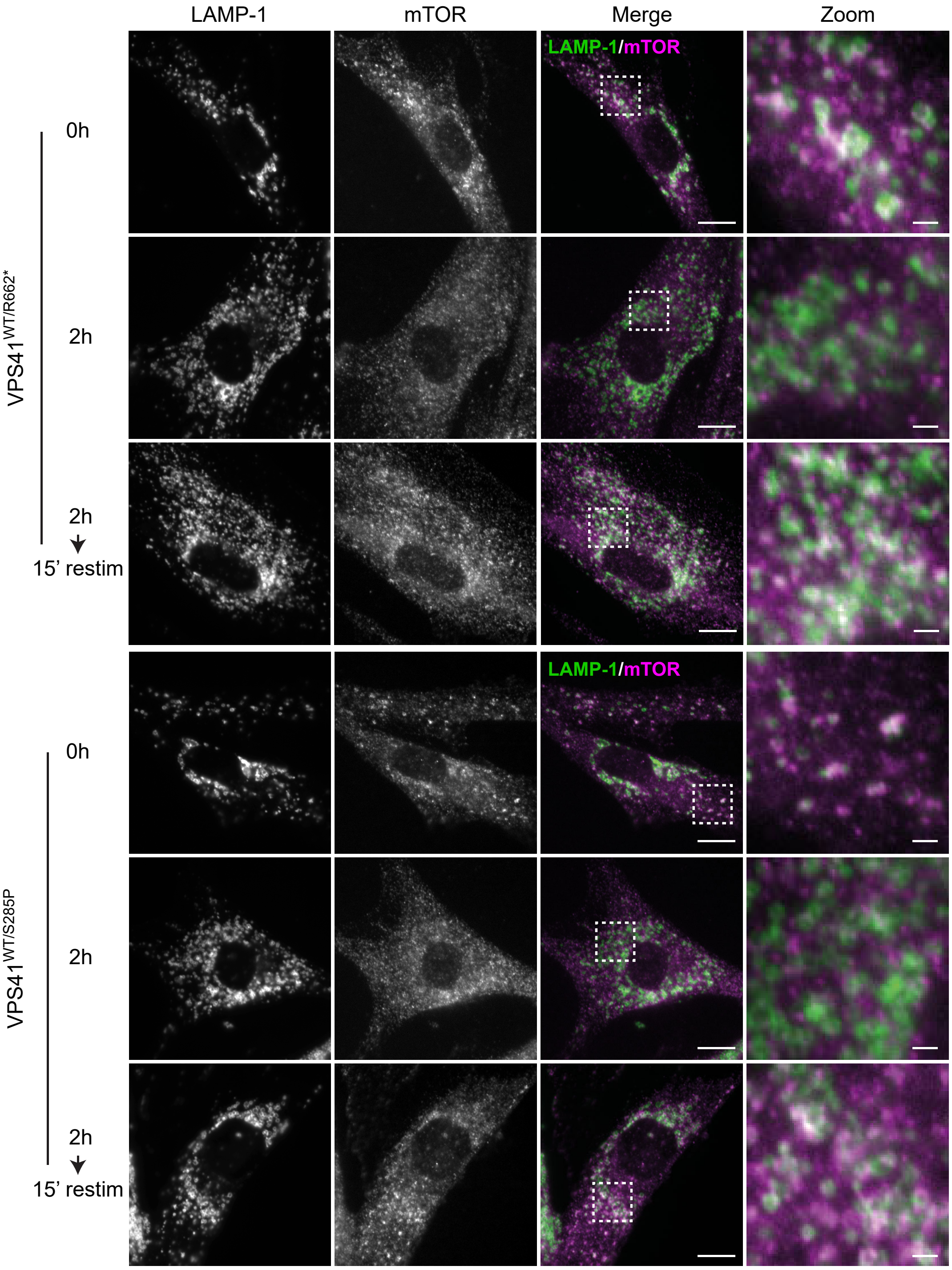

Appendix Figure S6. mTOR localization is not affected in parental fibroblasts.

Immunofluorescence of parental fibroblasts labeled for LAMP-1 and mTOR. In contrast to patient fibroblasts (Figure 5A), paternal *VPS41*$^{WT/S285P}$ and maternal *VPS41*$^{WT/R662*}$ fibroblasts show appropriate mTOR localization in the presence and absence of nutrients (2 hour starvation (2h)) and after restimulation (2h + 15’ restim.) (n=3). Bars, 10µm; zoom, 1µm.

Appendix Figure S7

A

Predicted Cleavage Site

actctctcagactgagctATGG

actctctcagactgagctATTGG
actctctcagactgagctATTTTGG

B

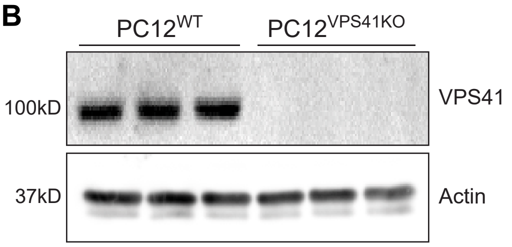

Appendix Figure S7. Validation of *VPS41* depletion in PC12 cells

A. Predicted Cas9 cleavage site for the generation of $PC12^{VPS41KO}$ cells.

B. Western blot analysis of PC12 cells to confirm depletion of VPS41 (n=3).

Appendix Figure S8

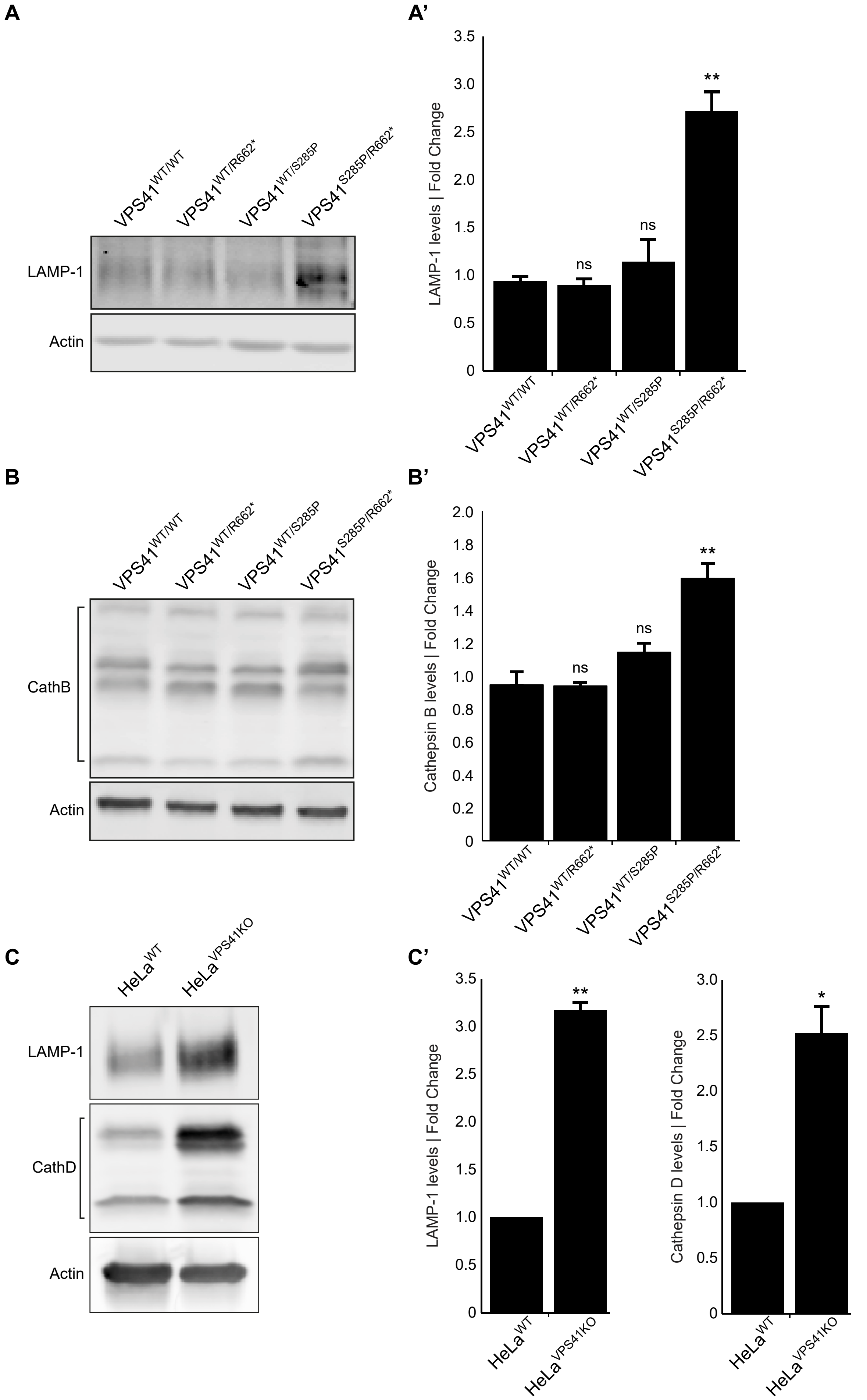

Appendix Figure S8. Mutation or depletion of *VPS41* results in increased expression of lysosomal proteins.

A/B. Fibroblasts. Western blot analysis shows a significant increase in LAMP-1 and Cathepsin B protein levels in $VPS41^{S285P/R662*}$ fibroblasts compared to $VPS41^{WT/WT}$, $VPS41^{WT/S285P}$ and $VPS41^{WT/R662*}$ fibroblasts (quantified in A′ and B′) (n=3).

C. HeLa cells. Western blot analysis shows a significant increase in LAMP-1 and Cathepsin D protein levels in $VPS41^{KO}$ cells compared to $HeLa^{WT}$ cells. The same $HeLa^{WT}$ and $HeLa^{VPS41KO}$ samples were analyzed in Figure 2B, showing the same actin control.

Data information: Data are represented as mean ± SEM. $*P < 0.05$, $**P < 0.01$. One-way ANOVA with Bonferroni correction (A′ and B′), or unpaired *t*-test (C′). Exact p-values are reported in Appendix Table S3.

Appendix Figure S9

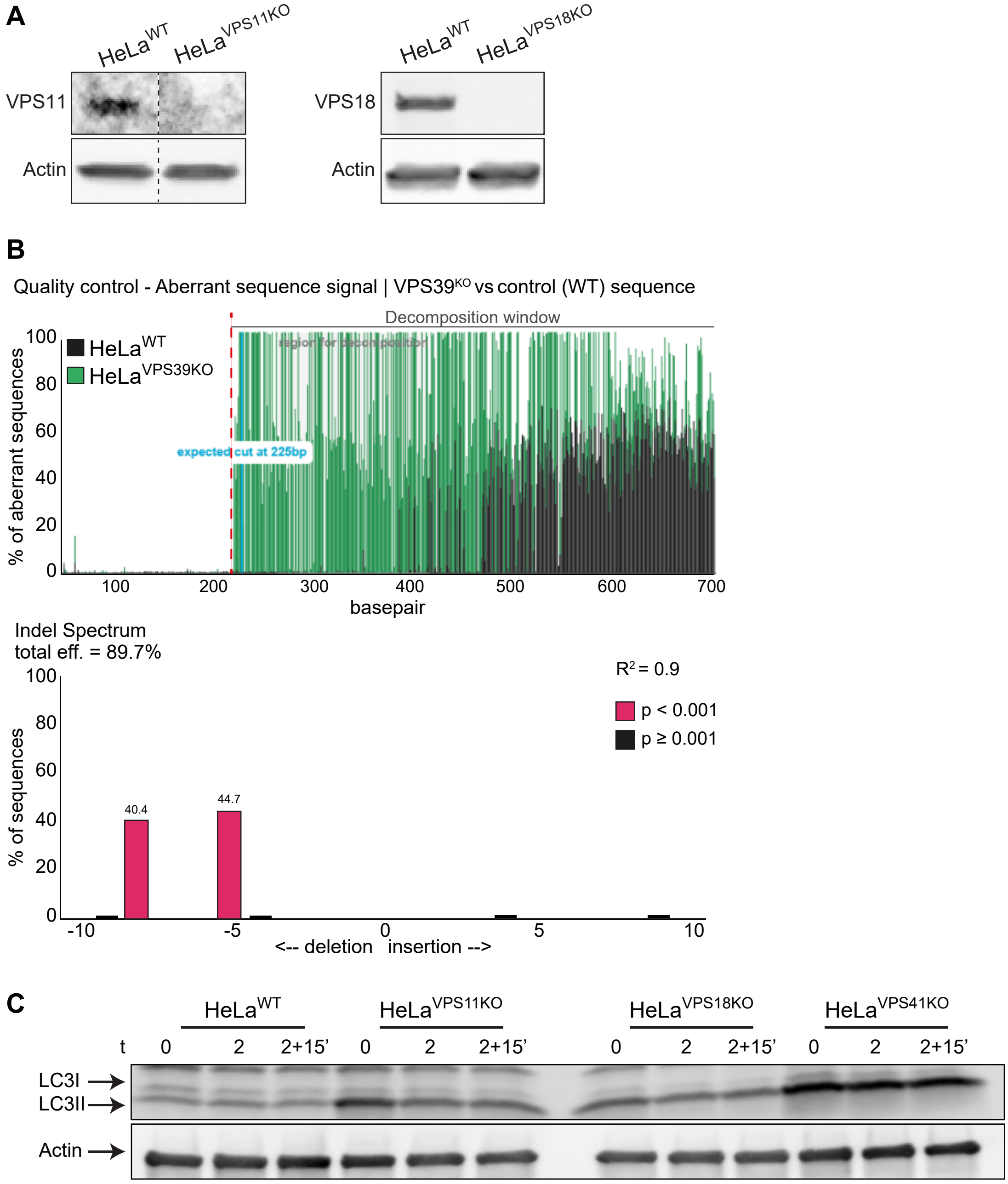

0= Steady state conditions
2= 2 hour starvation
2+15'= 2 hour starvation + 15 minutes restimulation

Appendix Figure S9. Depletion of HOPS subunits results in increased autophagosome formation.

A. Western blots confirming full knock-out of *VPS11* and *VPS18* in HeLa KO cells.

B. *VPS39* depletion in HeLa cells is confirmed using Tracking of Indels by Decomposition (TIDE) analysis. Aberrant signal sequence visualized for control (black) and HeLa^{VPS39KO} cells (green). The red dotted line represents the expected break site. Trace decomposition yields the spectrum of indels with their frequencies, visualized using an indel spectrum. The frequency spectrum indicates a 5 and 8 basepair deletion in VPS39 alleles, both resulting in frameshift.

C. Western blot of LC3 to determine autophagosome formation in HeLa^{VPS11KO}, HeLa^{VPS18KO} and HeLa^{VPS41KO} cells. All 3 KO cell lines show increased LC3II protein levels independent of nutrient availability (n=3). These data imply that absence of HOPS leads to increased LC3II levels.

Appendix Figure S10

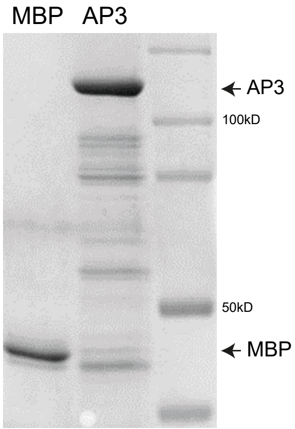

Appendix Figure S10. MBP-AP3 pulldown

Representative Coomassie stained gel of Maltose Binding Protein (MBP) and MBP-AP3 pulldown inputs.

Appendix Figure S11

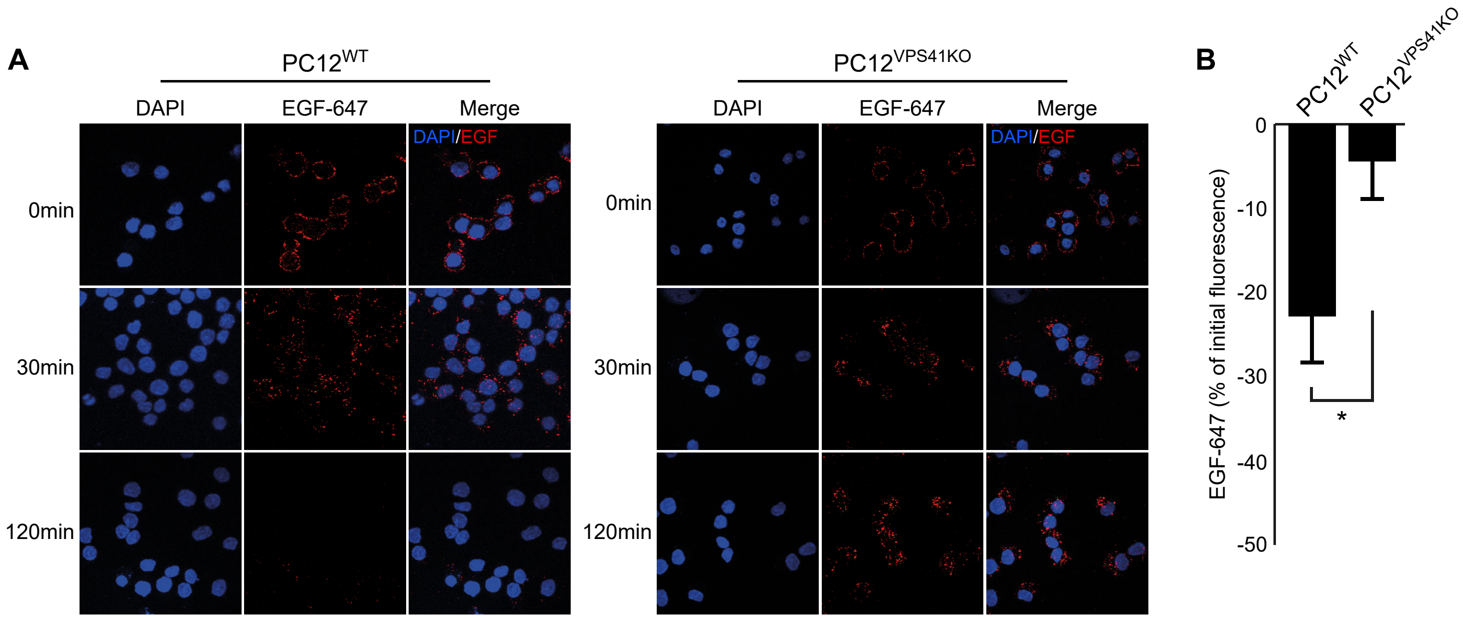

Appendix Figure S11. PC12^{VPS41KO} cells are impaired in degradation of endocytic cargo.

A. EGF-ALEXA647 (EGF-647) uptake and degradation in PC12^{WT} and PC12^{VPS41KO} cells. EGF-647 uptake was imaged at 0 minutes, 30 minutes and 120 minutes. PC12^{VPS41KO} cells show little loss of fluorescence, indicating a delay in delivery of EGF to enzymatically active compartments.

B. Measurement and quantification of EGF degradation at 2 hour time point using CyAn flow cytometry.

Data information: Data are represented as mean ± SEM. *$P < 0.05$. Unpaired *t*-test. Exact p-values are reported in Appendix Table S3.

Appendix Supplementary methods

Diagnostic sequencing techniques used in the patients

Whole Exome sequencing, filtering and variant analysis

Whole Exome sequencing (WES) was performed on genomic DNA extracted from whole blood from the affected children and their parents.

Family 1

Subjects 1 and 2 had clinical exome sequencing performed at GeneDx®. Exon targets were isolated by capture with IDT xGen Exome Research Panel v1.0 (Integrated DNA Technologies, Coralville, IA). The sequencing technology and variant interpretation protocol has been previously described (Retterer *et al,* 2015). The general assertion criteria for variant classification are publicly available on the GeneDx® ClinVar submission page (http://www.ncbi.nlm.nih.gov/clinvar/submitters/26957/).

Filtering for gene variants present in both the proband and brother with frequencies less than 0.01 in the gnomAD database and 0.001 in the GeneDx database revealed a total of 89 variants (manually subtracted variants with LowQ greater than 0). The rare variants are listed in Dataset EV1. Of these variants, we did not identify variants in established autosomal dominant disease genes that are absent in gnomAD, in established X-linked disease genes that have not been observed as hemizygous in gnomAD and no homozygous variants in established autosomal recessive disease genes. Compound heterozygous missense variants in the ITGB4 gene were identified (NM_001005731.2: c.3124G>A, NP_001005731.1 p.V1042M and NM_001005731.2 c.2512C>T, NP_001005731.1 p.R838W). However, ITGB4 is associated with autosomal recessive non-Herlitz junctional epidermolysis bullosa which is not a good phenotypic fit for this family. Both siblings are compound heterozygous for a paternal missense and a maternal nonsense in the *VPS41* gene. The *VPS41* missense (chr7:38816308 NM_014396.3: c.853T>C, NP_055211.2: p.S285P) has mixed in silico predictors and is observed in 2/282128 (0.0007%) alleles in gnomAD. The *VPS41* nonsense (chr7:38783140 NM_014396.3: c.1984C>T, NP_055211.2: p.R662*) is predicted to cause loss of normal protein function either through protein truncation or nonsense-mediated mRNA decay. The p.R662X variant is observed in 98/282180 (0.03%) alleles in gnomAD and no individuals are reported to be homozygous (see also Table 1 in main manuscript).

Family 2

Trio-based exome sequencing was performed following routine diagnostic procedures as described previously (Haer-Wigman *et al,* 2017). Essentially, DNA was sequenced on an Illumina HiSeq system after exome enrichment using the Agilent SureSelectXT Human All Exon 50Mb Kit version 4. Reads were aligned to the Hg19 reference genome with BWA and variants were called using GATK and annotated using an in-house developed pipeline. Filtering for gene variants was performed by filtering for variants in exonic and splice regions with frequencies lower than 0.01 in either the dbSNP, in-house or gnomAD databases. Variants that were de novo, homozygous or compound heterozygous are listed in Dataset EV2. The analysis resulted in reporting compound heterozygote mutations in the *VPS41* gene; a paternal canonical splice acceptor variant in intron 17 [NM_014396.3: c.1423-2A>G, r.spl? and a maternal nonsense mutation in the CHCR domain [NM_014396.3: c.1984C>T, NP_055211.2: p.Arg662*]. This patient also carried a homozygous mutation of unknown significance in UPF3A [NM_080687.2: c.707G>A, NP_542418.1: pArg236Gln] for which both parents and the healthy sibling were heterozygotes. UPF3A encodes the protein UPF3 regulator of nonsense transcripts homolog A, which has a role in the nonsense-mediated mRNA decay (NMD) complex. The variant is located in an evolutionary conserved nucleotide and amino acid, but not within a known functional domain of the protein. Thus far, variants in the gene have not been linked to human disease.

Sanger sequencing

Sanger sequencing was performed to test for the presence of the identified variants in *VPS41* and *UPF3A* in the healthy sibling of patient 3. Polymerase chain reaction (PCR) was performed prior to Sanger sequencing. PCR primers were designed by Primer3 software (http://bioinfo.ut.ee/primer3/). PCR amplification was performed using the AmpliTaq Gold 360 Master Mix (Invitrogen, Life Technologies, Carlsbad, CA, USA) according to the manufacturer's protocol. PCR fragments were purified with ExoSAP-IT (Affymetrix, Santa Clara, CA, USA) and subsequently sequenced on an ABI3730xl DNA Analyzer using BigDye Terminator v3.1 (Applied Biosystems, Foster City, CA, USA). Analysis of sequence data was performed using Mutation Surveyor (SoftGenetics, State College, PA, USA).

References to this supplement

Haer-Wigman L, Van Zelst-Stams WA, Pfundt R, Born LI van den, Klaver CW, Verheij JB, Hoyng CB, Breuning MH, Boon CJ, Kievit AJ *et al* (2017). Diagnostic exome sequencing in 266 Dutch patients with visual impairment. *Eur J Hum Genet* 25(5):591-599. doi:10.1038/ejhg.2017.9

Retterer K, Juusola J, Cho MT, Vitazka P, Millan F, Gibellini F, Vertino-Bell A, Smaoui N, Neidich J, Monoghan KG *et al* (2015). Clinical application of whole-exome sequencing across clinical indications. *Genet Med* 18(7):696-704. doi:10.1038/gim.2015.148

Appendix Table S1

Table S1. Mutation analysis on genes associated with X-linked mental retardation and dystonia as well as iron deposition in the basal ganglia were performed. No mutations were found.

	**Tested gene**
DYT1	TOR1A
Dystonia Myoclonus, DYT11	SGCE
FAHN, spastic paraplegia 35	FA2H
Infantile neuroaxonal dystophyl, Neurodegeneration with brain iron accumulation 2B	PLA2G6
Partington syndrome	ARX
Renpenning syndrome	PQBP1
Syndromic X-linked mental retardation, Claes-Jensen type	JARID1C
Mental retardation, X-linked 58	TM4SF2
Mental retardation, X-linked 63	FACL4
Mental retardation, X-linked 89	ZNF41
Mental retardation, X-linked 90	DLG3
Rett syndrome	MECP

Appendix Table S2

Table S2. Antibodies and reagents used.

**Primary Antibody**	**Use**	**Dilution**	**Company**	**Catalog Number**
M α LAMP-1 CD107a	IF	1:250	BD Pharmingen	555798
	WB	1:1000		
	EM (Tokuyasu)	1:1500		
M α LAMP-2	EM (Tokuyasu)	1:150	BD Pharmingen	555803
M α LC3	IF	1:100	Cosmo Bio Co., LTD	CTB-LC3-2-IC
R α LC3	IF	1:2000	Novus Biologicals	NB600-1384
	WB	1:1000		
R α p70 S6	WB	1:1000	Cell Signaling	9202
R α phospho-p70 S6 (Thr389)	WB	1:1000	Cell Signaling	9205
R α 4E BP1	WB	1:1000	Cell Signaling	9644
R α phospho-4E BP1 (Ser65)	WB	1:1000	Cell Signaling	9451
R α ULK1	WB	1:1000	Cell Signaling	8054
R α phospho-ULK1 (Ser757)	WB	1:1000	Cell Signaling	14202
G α Cathepsin B	WB	1:1000	RD Systems	AF953
	EM (Tokuyasu)	1:100		
G α Cathepsin D	IF	1:500	RD Systems	AF1014
	WB	1:2000		
M α VPS41	WB	1:500	Santa Cruz	SC-377271
M α Actin	WB	1:2000	MP Biomedicals	69100
M α GFP	WB	1:2000	Roche	11814460001
R α GFP	WB	1:1000	Invitrogen	A6455
R α FLAG	WB	1:2000	Sigma	F7429
R α V5	WB	1:1000	Sigma	V8137
	IF	1:400		
M α V5	WB	1:1000	Invitrogen	R960-25
	IF	1:200		
R α mTOR	IF	1:250	Cell Signaling	2983
R α TFE3	IF	1:250	Cell Signaling	14779
M α Hsp65	WB	1:2000	Gift from Van Eden	
R α VPS18	WB	1:500	Abcam	Ab178416
R α SgII	WB		Meridian Life Science	K55101R
R α His6	WB	1:2000	Cell Signaling	12698
G α HA	WB	1:2000	Genscript	A00168-100
R α TFEB	WB	1:1000	Cell Signaling	4240
R α VPS11	WB	1:500	Abcam	ab170869
**Secondary Antibody**	**Use**	**Dilution**	**Company**	**Catalog Number**

G α M – ALEXA568	IF	1:250	Thermofisher	A11031
G α M – ALEXA488	IF	1:250	Thermofisher	A11029
G α R – ALEXA568	IF	1:250	Thermofisher	A11036
D α R – ALEXA488	IF	1:250	Thermofisher	A21206
IRDye® 800CW G α R – IgG	WB	1:5000	LI-COR	925-32211
IRDye® 800CW G α M - IgG	WB	1:5000	LI-COR	925-32210
IRDye® 680RD G α R - IgG	WB	1:5000	LI-COR	925-68071
G α M – IgG – ALEXA680	WB	1:4000	Thermofisher	A28183
R α M IgG (bridging antibody)	EM (Tokuyasu)	1:200	Rockland	610-4120
D = Donkey, G = Goat, M = Mouse, R = Rabbit				

**Reagents**	**Use**	**Dilution**	**Company**	**Catalog Number**
Protein-A gold 10 or 15nm	EM (Tokuyasu)		Cell Microscopy Core, UMC Utrecht	
BSA-gold 5nm	Endocytic uptake/EM		Cell Microscopy Core, UMC Utrecht	
10.000MW Dextran-ALEXA568	Endocytic uptake/IF	1:100	Invitrogen	D22912
Lysotracker™ Red	Lysosomal pH/IF	1:100	Thermofisher	L7528
SiR-lysosome	CathepsinD activity/IF	1:1000	Spirochrome	SC012
MagicRed CathepsinB	CathepsinB activity/IF	1:260	Abcam	Ab270772
EGF-biotin	EGF degradation/IF	5µg/ml	Goldbio	1150-04
Streptavidin647	EGF degradation/IF	1:5 ratio w/EGF	Thermofisher	S21374
Prolong Diamond w/ DAPI	Antifade/IF		Thermofisher	P36971
MG132	Proteasome inhibitor	50µM	Sigma	474787
Bafilomycin A1	v-ATPase inhibitor	100nM	Sigma	19-148
Torin-1	mTOR inhibitor	250nM	Selleckchem	S2827

Appendix Table S3

Table S3. Summary of statistical tests and p values.

Figure	Test used	Comparison	*p* value
Fig 2A′	One-way ANOVA analysis with Tukey's correction	WT/WT vs. WT/S285P	0.0473
		WT/WT vs. S285P/R662*	<0.0001
		WT/WT vs. c.1423-2A>G/R662*	<0.0001
		WT/R662* vs. WT/S285P	0.0237
		WT/R662* vs. S285P/R662*	<0.0001
		WT/S285P vs. S285P/R662*	0.024
Fig 2C′	Unpaired t-test	WT vs. $VPS41^{KO}$	0.0024
Fig 2E	One-way ANOVA analysis with Tukey's correction	WT/WT vs. S285P/R662*	<0.0001
		WT/R662* vs. S285P/R662*	0.0171
		WT/S285P vs. S285P/R662*	0.0017
Fig 2F		WT/WT vs. S285P/R662*	<0.0001
		WT/R662* vs. S285P/R662*	0.0026
		WT/S285P vs. S285P/R662*	0.0071
Fig 3A′	Unpaired t-test	WT/WT vs. S285P/R662*	0.0223
		WT/WT vs. c.1423-2A>G/R662*	0.0007
Fig 3E	One-way ANOVA analysis with Bonferroni correction	$VPS41^{KO}$-EV vs. $VPS41^{KO}$-WT	0.0009
Fig 3F	One-way ANOVA analysis with Tukey's correction	WT/WT vs. WT/R662*	0.0186
		WT/WT vs. S285P/R662*	0.0126
Fig 4A′	Unpaired t-test	WT/WT vs. S285P/R662*; 0h	0.0085
		WT/WT vs. S285P/R662*; 2h	0.0465
Fig 4B′	One-way ANOVA analysis with Tukey's correction	WT/WT; 0h vs. 2h	<0.0001
		S285P/R622*; 0h vs. 2h	<0.0001
		WT/WT vs. S285P/R662*; 0h	<0.0001
		WT/WT vs. S285P/R662*; 2h	0.02244
Fig 4C′	One-way ANOVA analysis with Bonferroni correction	WT; 0h vs. 2h	0.0043
		WT; 2h vs. 2h + 15min	0.0294
		WT vs. $VPS41^{KO}$; 0h vs. 0h	<0.0001
Fig 4D′	One-way ANOVA analysis with Bonferroni correction	$VPS41^{KO}$-EV vs. $VPS41^{KO}$-WT; 0h vs. 0h	0.0002
		$VPS41^{KO}$-WT; 0h vs. 2h	0.0037
Fig 4E′	Unpaired t-test	$VPS41^{KO}$-EV vs. $VPS41^{KO}$-WT	6.5390E-09
Fig 5A′	One-way ANOVA analysis with Bonferroni correction	WT/WT; 0h vs. 2h	0.0007
		WT/WT; 2h vs. 2h + 15min	0.0002
		WT/R662*; 0h vs. 2h	0.0329
		WT/R662*; 2h vs. 2h + 15min	0.0048

		WT/S285P; 0h vs. 2h	0.0498
		WT/S285P; 2h vs. 2h + 15min	0.0076
Fig 5B′	One-way ANOVA analysis with Bonferroni correction	WT/WT; 0h vs. 2h	0.0042
		WT/WT; 2h vs. 2h + 15min	<0.0001
		WT/R662*; 0h vs. 2h	0.0134
		WT/R662*; 2h vs. 2h + 15min	0.0002
		WT/S285P; 0h vs. 2h	0.0410
		WT/S285P; 2h vs. 2h + 15min	0.0002
Fig 5C′	One-way ANOVA analysis with Bonferroni correction	WT; 0h vs. 2h	<0.0001
		WT vs VPS41^{KO}; 0h vs. 0h	<0.0001
Fig 5D′	One-way ANOVA analysis with Bonferroni correction	VPS41^{KO}-WT; 2h vs. 2h + 15min	0.0004
Fig 5E′	Unpaired t-test	WT vs VPS41^{KO}; NT	0.0422
Fig 6C	One-way ANOVA analysis	VPS41^{KO} vs. VPS41^{KO}-WT	0.0053
		VPS41^{KO} vs. VPS41^{KO}-S285P	0.0140
Fig 6D	One-way ANOVA analysis	VPS41^{KO}; Basal vs. Stimulated	0.00875
		VPS41^{KO}-WT; Basal vs. Stimulated	0.00044
		VPS41^{KO}-S285P; Basal vs. Stimulated	0.00016
		VPS41^{KO} vs. VPS41^{KO}-WT; Stimulated	0.0452
Fig 7C	One-way ANOVA analysis with Tukey’s correction	Day 7; nontrans vs. WT/S285P	0.0022
		Day 7; nontrans vs. WT/R662*	0.0071
		Day 7 vs. Day 10 transfected	<0.0001
		Day 10; nontrans vs. S285P/R662*	0.0407
Fig EV3B	One-way ANOVA analysis with Tukey’s correction	WT/NT/BafA1 vs. VPS41^{KO}/NT/BafA1	0.0021
		WT/ST/BafA1 vs. VPS41^{KO}/ST/BafA1	0,0006
		WT/15′/BafA1 vs. VPS41^{KO}/15′/BafA1	<0.0001
Fig EV3C	Unpaired t-test	WT vs. VPS41^{KO}; NT	4.9953E-06
Fig EV4D	One-way ANOVA analysis with Bonferroni correction	WT vs. VPS41^{KO}	<0.0001
		VPS41^{KO} vs. VPS41^{KO}-WT	<0.0001
		VPS41^{KO} vs. VPS41^{KO}-R662*	0.0205
Fig EV5A′	One-way ANOVA analysis with Bonferroni correction	WT vs. VPS11^{KO}	<0.0001
		WT vs. VPS18^{KO}	<0.0001
		WT vs. VPS39^{KO}	<0.0001
		WT vs. VPS41^{KO}	0.0006

Fig S1A′	One-way ANOVA analysis with Bonferroni correction	WT/WT vs. S285P/R662*	0.0079
Fig S1B′	Unpaired t-test	WT vs. $VPS41^{KO}$	0.0056
Fig S2A	One-way ANOVA analysis with Tukey’s correction	WT/WT vs. S285P/R662*	<0.0001
		WT/R662* vs. S285P/R662*	0.0426
		WT/S285P vs. R662*	0.0067
Fig S3B	One-way ANOVA analysis with Tukey’s correction	WT/WT vs. WT/R662*	0.0019
		WT/WT vs. S285P/R662*	0.0005
		WT/R662* vs. WT/S285P	0.0228
		WT/S285P vs. S285P/R662*	0.0066
Fig S8A′	One-way ANOVA analysis with Bonferroni correction	WT/WT vs. S285P/R662*	0.0266
Fig S8B′	One-way ANOVA analysis with Bonferroni correction	WT/WT vs. S285P/R662*	0.0099
Fig S8C′	Unpaired t-test	WT vs. $VPS41^{KO}$; LAMP-1	0.0014
		WT vs. $VPS41^{KO}$; Cathepsin D	0.0228
Fig S11B	Unpaired t-test	WT vs. $VPS41^{KO}$	0.0227
