# Supplementary material for: Neurodegenerative VPS41 variants inhibit HOPS function and mTORC1‐dependent TFEB/TFE3 regulation
Source: EMBO Mol Med. 2021 Apr 14;13(5):e13258. doi: 10.15252/emmm.202013258 (PMC8103106; doi:10.15252/emmm.202013258)
Supplement: Supplementary file 2 — Expanded View Figures PDF [file EMMM-13-e13258-s006.pdf]

Expanded View Figures

Figure EV1. Mutations in *VPS41* cause a neurodegenerative disease.

A Pedigrees of family 1 affected by recessive mutations in *VPS41*. Circle = female, square = male, black-filled shape = individual phenotypically affected.
B Head circumference of patients 1 (green), 2 (yellow), and 3 (red) in cm.
C Outline of the VPS41 protein depicting distinct domains. The WD40 domain facilitates protein–protein interactions and the CHCR and RING domains enable homo-oligomerization and are required for HOPS complex formation and regulated secretion. Mutations in *VPS41* were identified using whole exome sequencing. The two siblings (patients 1 and 2) bear compound heterozygous mutations; a missense mutation $VPS41^{S285P}$ in the WD40 domain and a nonsense mutation $VPS41^{R662*}$ at the C-terminus resulting in a premature stop codon. Patient 3 bears a canonical splicing variant expected to destroy an acceptor site ($VPS41^{c.1423\text{-}2A>G}$) in the TPR-like domain and shares the nonsense mutation ($VPS41^{R662*}$) at the C terminus with the other patients.
D Pedigree of family 2 affected by recessive mutations in *VPS41*. Circle = female, square = male, black-filled shape = individual phenotypically affected, double line = consanguinity.
E Patient 3 developed coarse facial features with heavy eyebrows, gingival hypertrophy, protruding tongue, thick lips, and thick ear lobes (I; age 1 year, 7 months, II and III; age 4 years, 10 months).
F A spinal X-ray showed a kyphosis at C3 and hypoplastic distal phalanges on digits 2, 3, and 5 and a short metacarpal 1, bilaterally (IV; age 5 years, V; age 11 years).

© 2021 The Authors

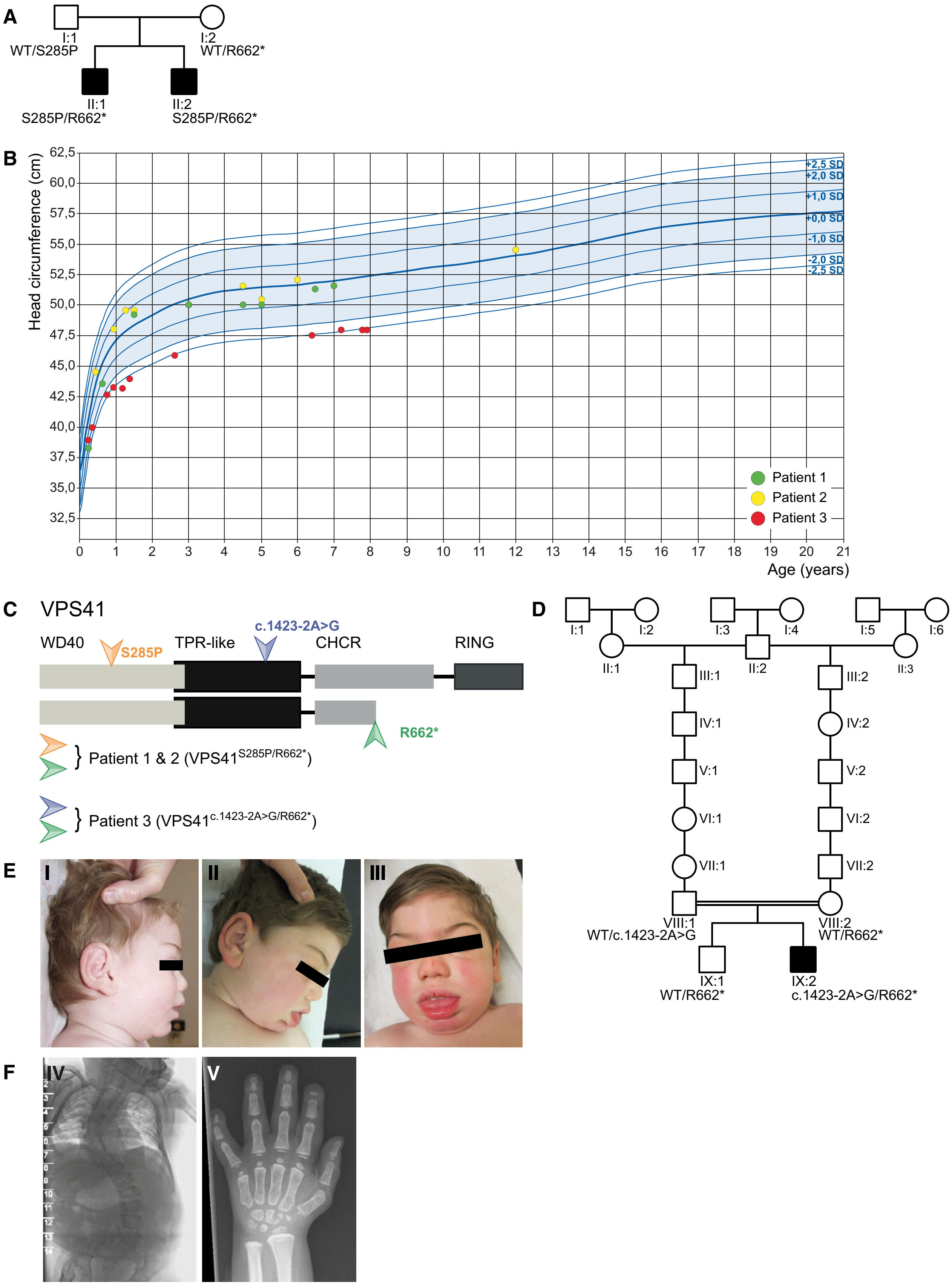

Figure EV1.

© 2021 The Authors

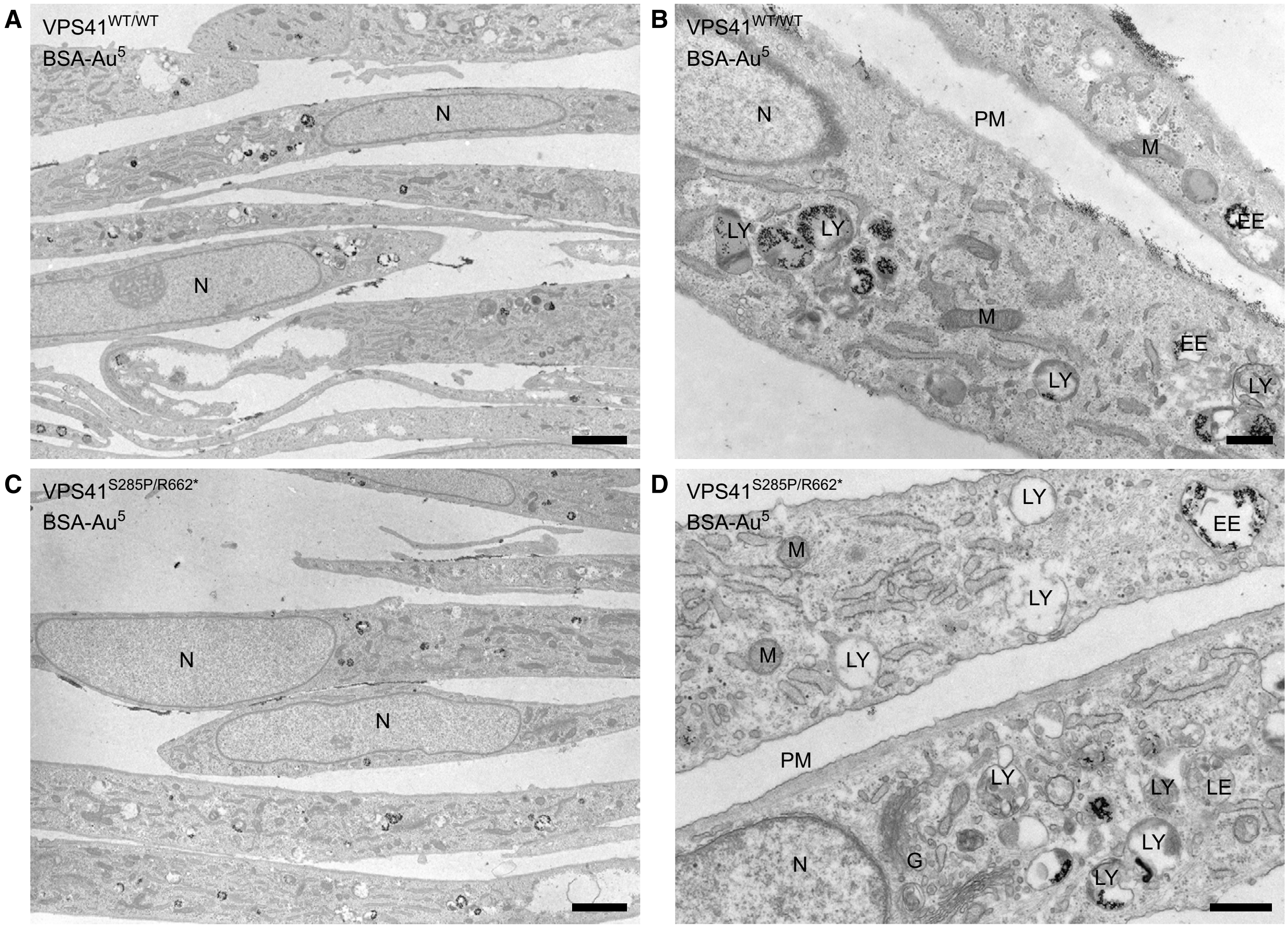

Figure EV2. Mutations in *VPS41* do not cause a lysosomal storage phenotype.

A, B Electron micrographs of Epon sections of *VPS41*$^{WT/WT}$ fibroblasts.

C, D Electron micrographs of Epon sections of *VPS41*$^{S285P/R662*}$ fibroblasts.

Data information: Cells were incubated with BSA-Au^{5} for 2 h to label endolysosomal compartments. Both primary fibroblast cell lines show a high variation in the appearance of endolysosomal compartments. There is no aberrant swelling of endolysosomal organelles in patient *VPS41*$^{S285P/R662*}$ fibroblasts. G = Golgi, EE = Early endosome, LE = Late endosome, LY = Lysosome, M = Mitochondria, N = Nucleus, PM = Plasma membrane. Scale bars low magnification (A, C), 2 μm; High magnification (B, D), 500 nm.

© 2021 The Authors

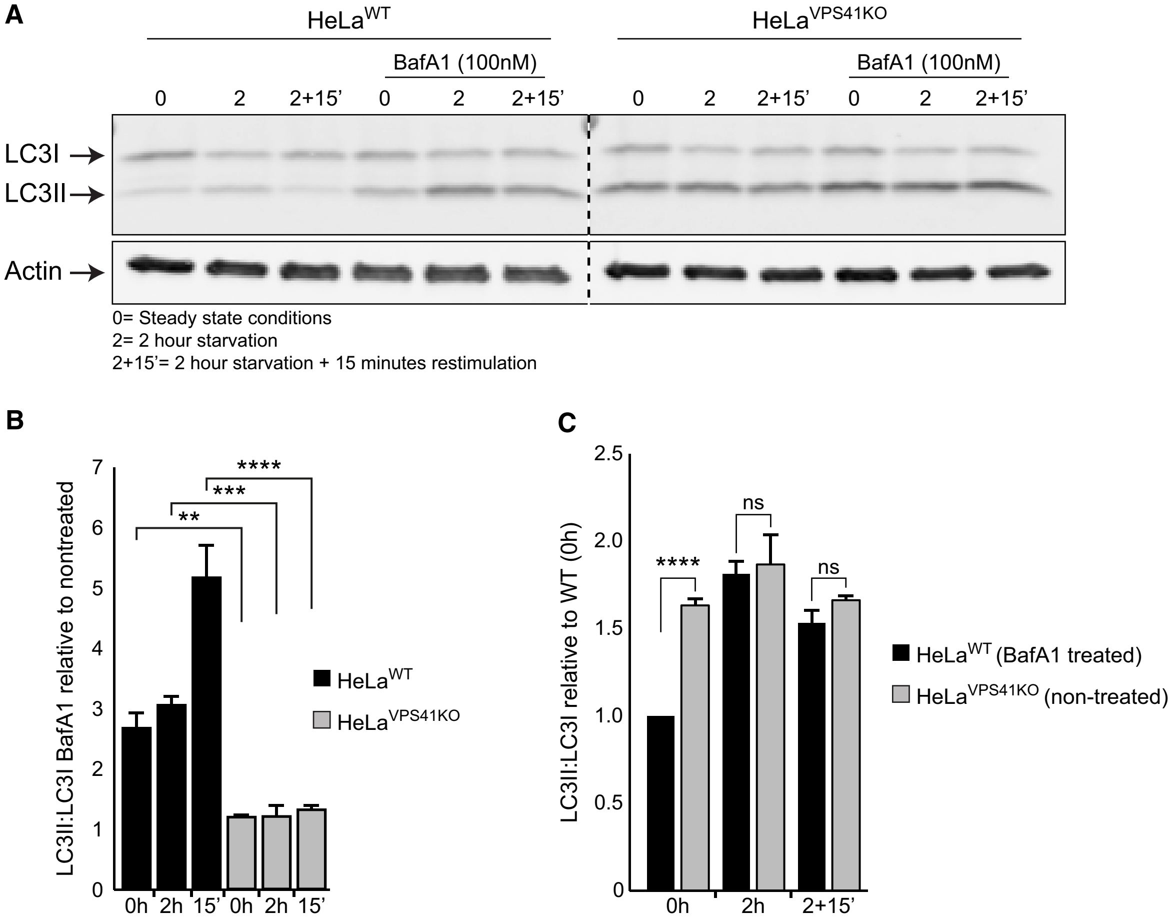

Figure EV3. *VPS41^{KO}* cells are insensitive to Bafilomycin A1 treatment.

A Western blot of LC3 protein levels in HeLa^{WT} and HeLa^{VPS41KO} cells with and without Bafilomycin A1 (BafA1) treatment. Cells depleted of *VPS41* show increased LC3II protein levels compared with WT cells. BafA1 treatment increases LC3II protein levels in HeLa^{WT} cells, whereas HeLa^{VPS41KO} cells are relatively insensitive ($n = 2$).

B Quantification of LC3II:LC3I levels. Per cell line and per condition the increase in LC3II:LC3I after BafA1 treatment are plotted against non-treated cells. In contrast to HeLa^{WT} cells, the HeLa^{VPS41KO} cells show only a minor increase ($n = 2$).

C Quantification of LC3II:LC3I levels in HeLa^{WT} BafA1 treated compared with non-treated HeLa^{VPS41KO} cells in steady state (0 h), starved (2 h) and refeeding (2-h starvation followed by 15-min restimulation) conditions. Similar LC3II ratios are observed after starvation and BafA1 treatment of HeLa^{WT} cells, indicating that the elevated LC3 levels in HeLa^{VPS41KO} cells are caused by increased autophagic induction and decreased autophagic flux ($n = 2$).

Data information: Data are represented as mean ± SD. $^{**}P < 0.01$, $^{***}P < 0.001$, $^{****}P < 10^{-5}$. One-way ANOVA analysis with Tukey's correction (B) or unpaired *t*-test (C). Exact *P*-values are reported in Appendix Table S3.
Source data are available online for this figure.

 © 2021 The Authors

Figure EV4. *VPS41* deficiency causes decreased lysosomal localization of mTORC1 and defects in TFE3/TFEB signaling.

A *VPS41*$^{c.1423\text{-}2A>G/R662*}$ fibroblasts show constitutive nuclear localization of TFE3. Scale bars, 10 μm.
B HeLa^{WT} and HeLa^{VPS41KO} cells labeled for LAMP-1 and mTOR immunofluorescence. In HeLa^{VPS41KO} cells, mTOR is dissociated from LAMP-1-positive compartments regardless of nutrient state. In HeLa^{WT} cells, mTOR colocalizes with LAMP-1 in steady state (0 h) and after nutrient restimulation (2-h starvation followed by 15-min restimulation). Scale bars, 10 μm; zoom 1 μm.
C Rescue experiment of mTOR localization on cathepsin D-positive lysosomes in HeLa^{VPS41KO} cells transfected with VPS41^{WT}-APEX2-V5, VPS41^{S285P}-APEX2-V5, or VPS41^{R662*}-APEX2-V5. Cells were starved for 2 h and restimulated with full medium for 15 min. Scale bars, 10 μm; zoom, 1 μm.
D Quantification of mTOR colocalization with cathepsin D in rescued *VPS41*^{KO} cells. Reintroducing VPS41^{WT} results in increased colocalization of mTOR with lysosomes. > 15 cells per condition were quantified in this assay ($n = 2$).
E Immunofluorescence of 300 nm thick cryosections of PC12^{WT} and PC12^{VPS41KO} cells labeled for TFE3. PC12^{VPS41KO} cells show constitutive nuclear localization of TFE3. Scale bar, 10 μm.
F Western blots of TFEB. In HeLa^{WT} cells, molecular weight shifts are indicative for (de)phosphorylation events dependent on nutrient availability. mTORC1 inhibitor Torin-1 impairs TFEB phosphorylation and no shift in molecular weight is seen. In *VPS41*^{KO} cells, no molecular weight shift of TFEB is seen either, indicating impaired phosphorylation of TFEB.

Data information: Data are represented as mean ± SEM. *$P < 0.05$, ****$P < 10^{-5}$. One-way ANOVA analysis with Bonferroni correction. Exact *P*-values are reported in Appendix Table S3.
Source data are available online for this figure.

© 2021 The Authors

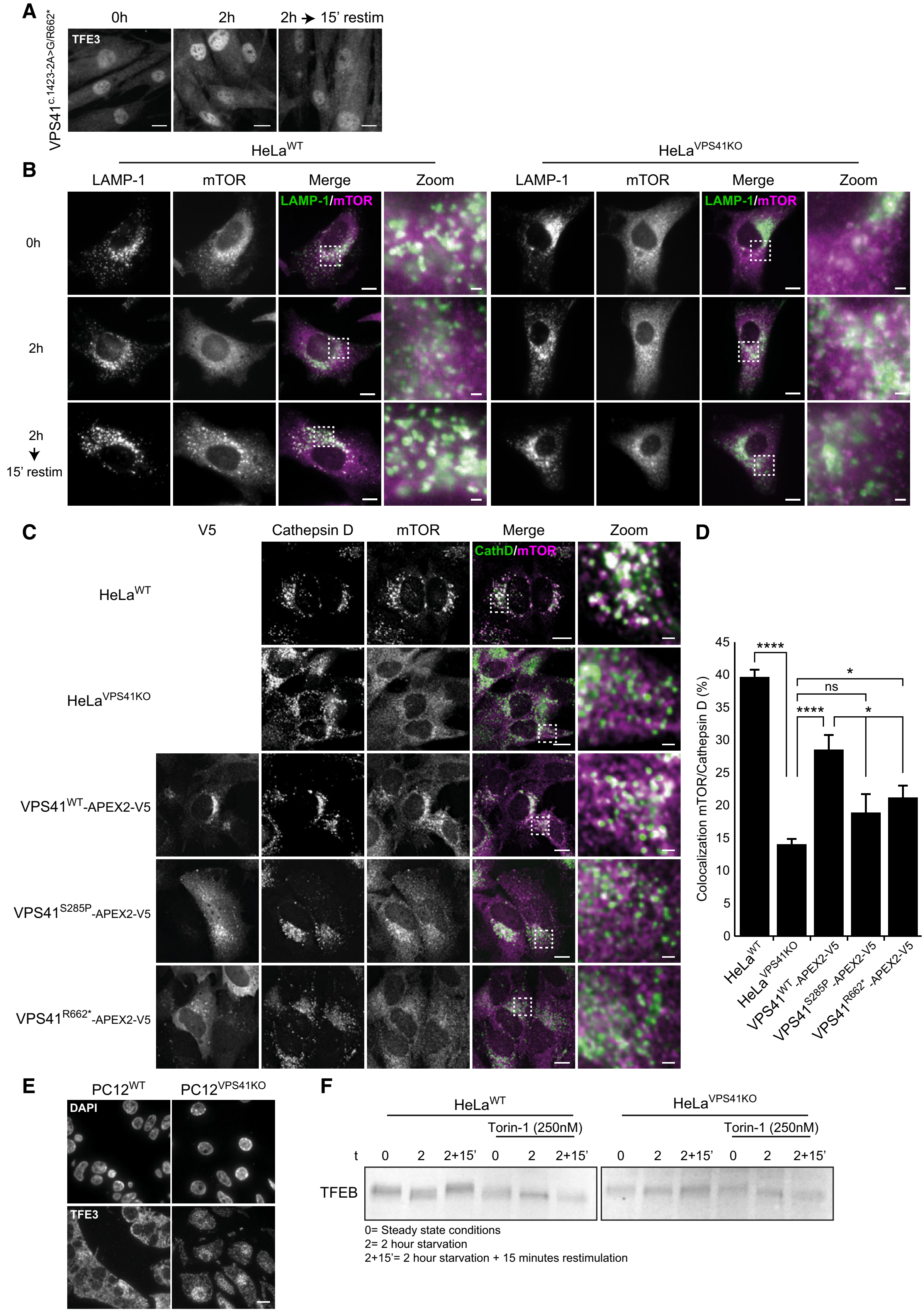

Figure EV4.

© 2021 The Authors

Figure EV5. The HOPS complex is required for mTORC1-dependent TFE3 regulation.

A Immunofluorescence microscopy of HeLa^{WT}, HeLa^{VPS11KO}, HeLa^{VPS18KO}, HeLa^{VPS39KO}, and HeLa^{VPS41KO} cells labeled for cathepsin D. All KO cells lines show more cathepsin D-positive compartments (quantified in A'). > 56 Cells per cell line were quantified ($n = 3$). Scale bars, 10 μm.

B Immunofluorescence microscopy of HeLa^{VPS11KO} and HeLa^{VPS18KO} cells labeled for TFE3 at steady state (0 h), starved (2 h), or restimulated (2-h starvation followed by 15-min restimulation) conditions. In both HeLa^{VPS11KO} and HeLa^{VPS18KO} cells, TFE3 is present in the nucleus regardless of nutrient availability, indicating that mTORC1-dependent regulation of TFE3 is impaired in cells depleted of HOPS subunits (quantified in B') ($n = 3$). Scale bars, 10 μm.

C Western blot of phosphorylated mTORC1 substrates S6K1 and 4EBP1 after starvation (2 h) and restimulation with full medium for 10, 30, or 60 min in HeLa^{VPS18KO} and HeLa^{VPS39KO} cells. After 30-min restimulation, both substrates are phosphorylated in both cell lines. These data show that phosphorylation of S6K1 and 4EBP1 is independent of the HOPS complex.

D Western blot showing ULK1, a substrate of mTORC1 involved in autophagy initiation, and phosphorylated upon nutrient availability. HeLa^{WT}, HeLa^{VPS18KO}, HeLa^{VPS39KO}, and HeLa^{VPS41KO} cell lines show an appropriate response in (de)phosphorylation of ULK1 upon starvation (2 h) and restimulation (2-h starvation followed by 30-min restimulation).

Data information: Data are represented as mean ± SEM. ***$P < 0.001$, ****$P < 10^{-5}$. One-way ANOVA analysis with Bonferroni correction. Exact P-values are reported in Appendix Table S3.
Source data are available online for this figure.

© 2021 The Authors

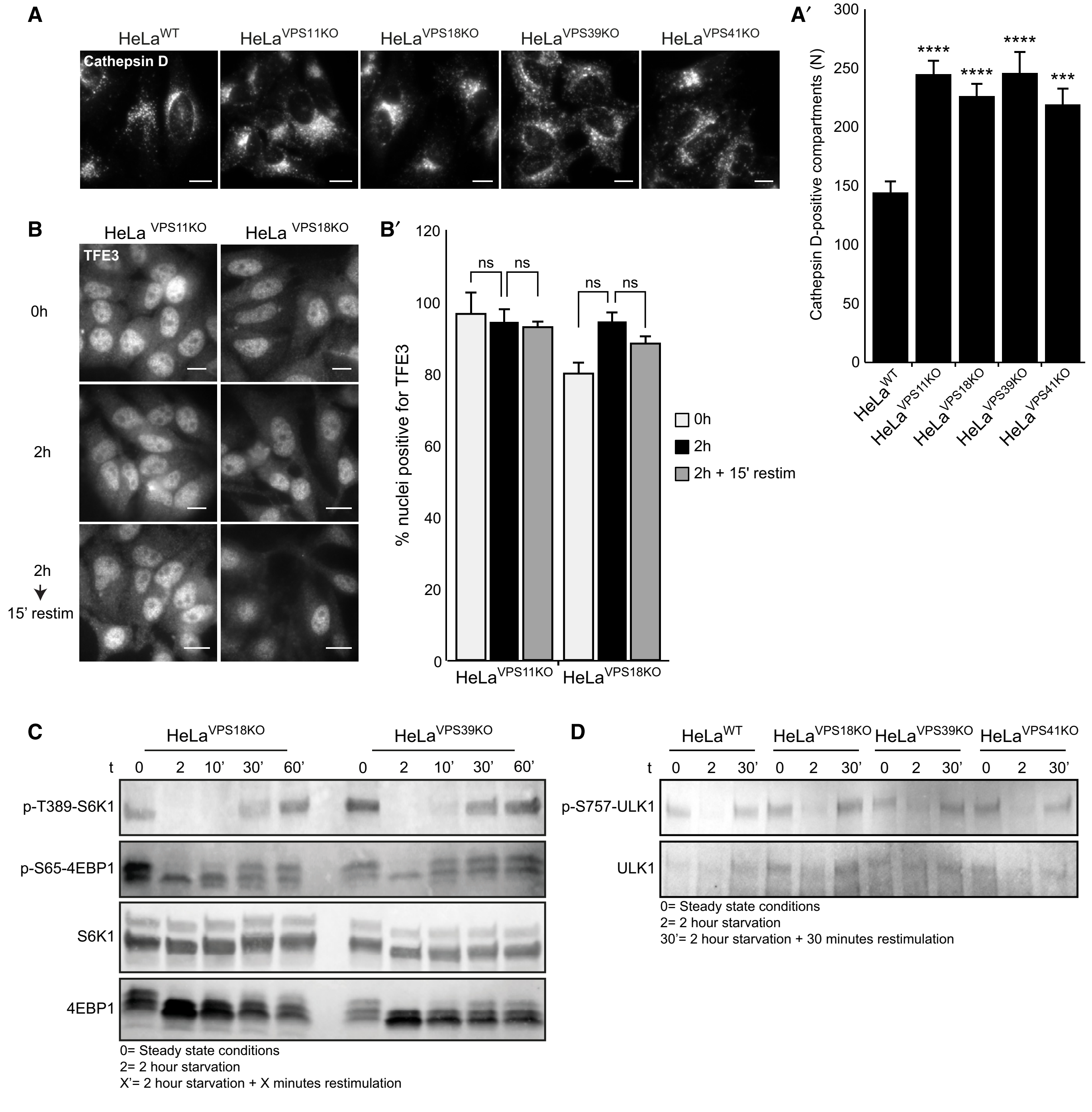

Figure EV5.

 © 2021 The Authors
