# Supplementary material for: Neurodegenerative VPS41 variants inhibit HOPS function and mTORC1‐dependent TFEB/TFE3 regulation
Source: EMBO Mol Med. 2021 Apr 14;13(5):e13258. doi: 10.15252/emmm.202013258 (PMC8103106; doi:10.15252/emmm.202013258)
Supplement: Supplementary file 3 — Dataset EV1 [file EMMM-13-e13258-s002.zip › Dataset_EV1_legend.docx]

**Dataset EV1**

The rare variants identified in patient 1. Of these variants, we did not identify variants in established autosomal dominant disease genes that are absent in gnomAD, in established X-linked disease genes that have not been observed as hemizygous in gnomAD and no homozygous variants in established autosomal recessive disease genes.
