# Supplementary material for: Neurodegenerative VPS41 variants inhibit HOPS function and mTORC1‐dependent TFEB/TFE3 regulation
Source: EMBO Mol Med. 2021 Apr 14;13(5):e13258. doi: 10.15252/emmm.202013258 (PMC8103106; doi:10.15252/emmm.202013258)
Supplement: Supplementary file 4 — Dataset EV2 [file EMMM-13-e13258-s010.zip › Dataset_EV2_legend.docx]

**Dataset EV2**

Variants that were de novo, homozygous or compound heterozygous, identified in patient 3.
