# Supplementary material for: Neurodegenerative VPS41 variants inhibit HOPS function and mTORC1‐dependent TFEB/TFE3 regulation
Source: EMBO Mol Med. 2021 Apr 14;13(5):e13258. doi: 10.15252/emmm.202013258 (PMC8103106; doi:10.15252/emmm.202013258)
Supplement: Supplementary file 5 — Movie EV1 [file EMMM-13-e13258-s001.zip › Movie_EV1_legend.docx]

**Movie EV1**

Patient 1 examined at age 20: he has global developmental delay with repetitive behaviors, pervasive tendencies and some obsessive behaviors. Video also shows bilateral limb dysmetria, bilateral flat feet with a mild feet dorsiflexion, generalized hypotonia and ataxic gait.
