# Supplementary material for: Neurodegenerative VPS41 variants inhibit HOPS function and mTORC1‐dependent TFEB/TFE3 regulation
Source: EMBO Mol Med. 2021 Apr 14;13(5):e13258. doi: 10.15252/emmm.202013258 (PMC8103106; doi:10.15252/emmm.202013258)
Supplement: Supplementary file 6 — Movie EV2 [file EMMM-13-e13258-s005.zip › Movie_EV2_legend.docx]

**Movie EV2**

Patient 2 examined at age 12.5, before DBS: he has generalized dystonia involving his face, tongue, neck, trunk and four limbs.
