# Supplementary material for: Neurodegenerative VPS41 variants inhibit HOPS function and mTORC1‐dependent TFEB/TFE3 regulation
Source: EMBO Mol Med. 2021 Apr 14;13(5):e13258. doi: 10.15252/emmm.202013258 (PMC8103106; doi:10.15252/emmm.202013258)
Supplement: Supplementary file 7 — Movie EV3 [file EMMM-13-e13258-s009.zip › Movie_EV3_legend.docx]

**Movie EV3**

Patient 2 examined 1.5 years after DBS: a notable improvement of trunk, head and arm dystonia, allowing him to sit straight and gain fine motor skills with both hands, is noticeable. By contrast, lower limbs dystonia is only slightly improved.
