# Supplementary figures and images for: Neurodegenerative VPS41 variants inhibit HOPS function and mTORC1‐dependent TFEB/TFE3 regulation
Source: EMBO Mol Med. 2021 Apr 14;13(5):e13258. doi: 10.15252/emmm.202013258 (PMC8103106; doi:10.15252/emmm.202013258)

Appendix Figure S4A

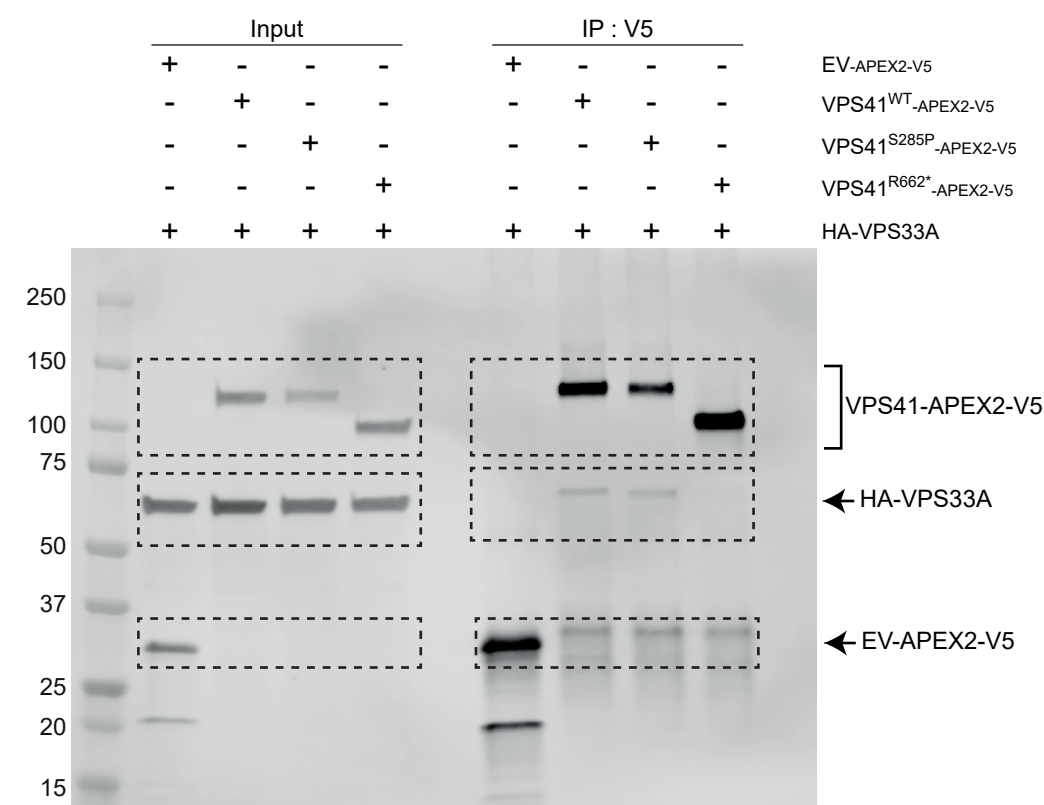

Appendix Figure S4B

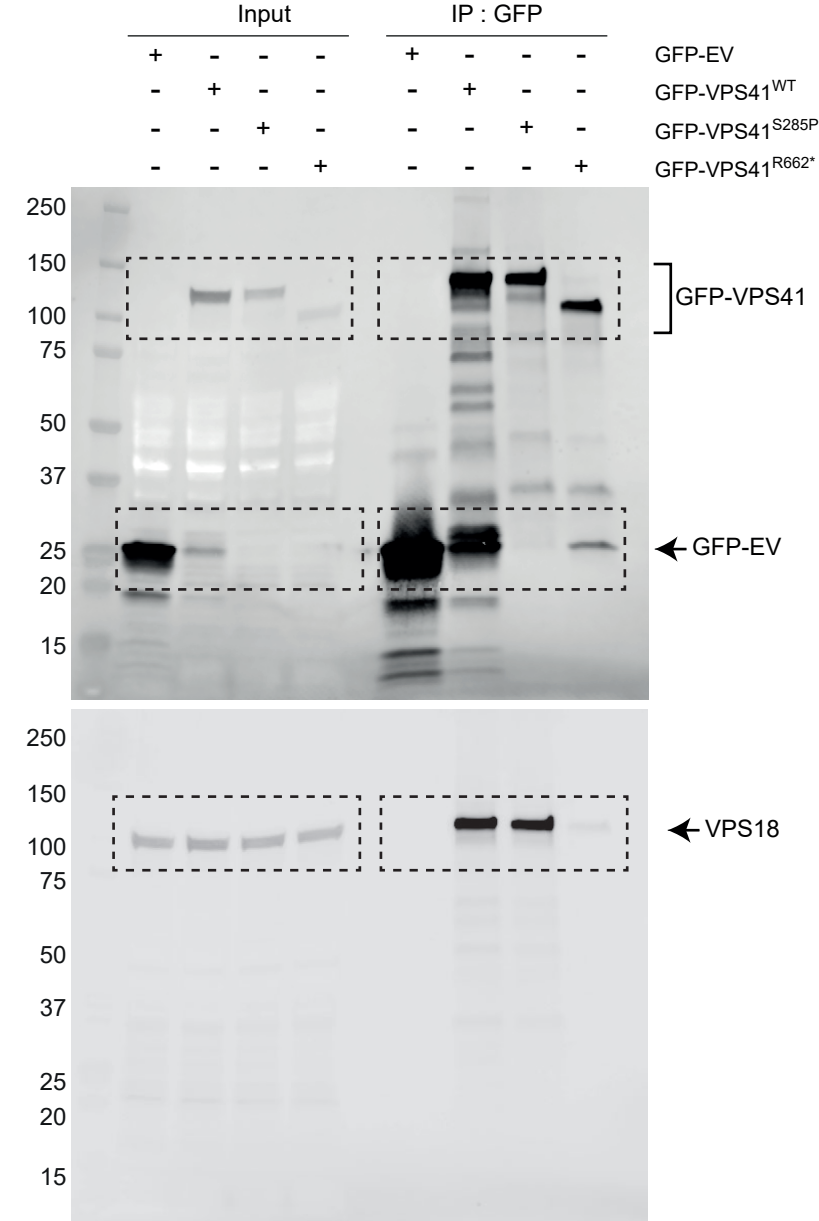

Supplement: Supplementary file 8 — Source Data for Expanded View and Appendix [file EMMM-13-e13258-s013.zip › EV-appendix-SD/EMM-2020-13258-V4_Source_Data_Appendix_Figure_S4.pdf]

Appendix Figure S9C

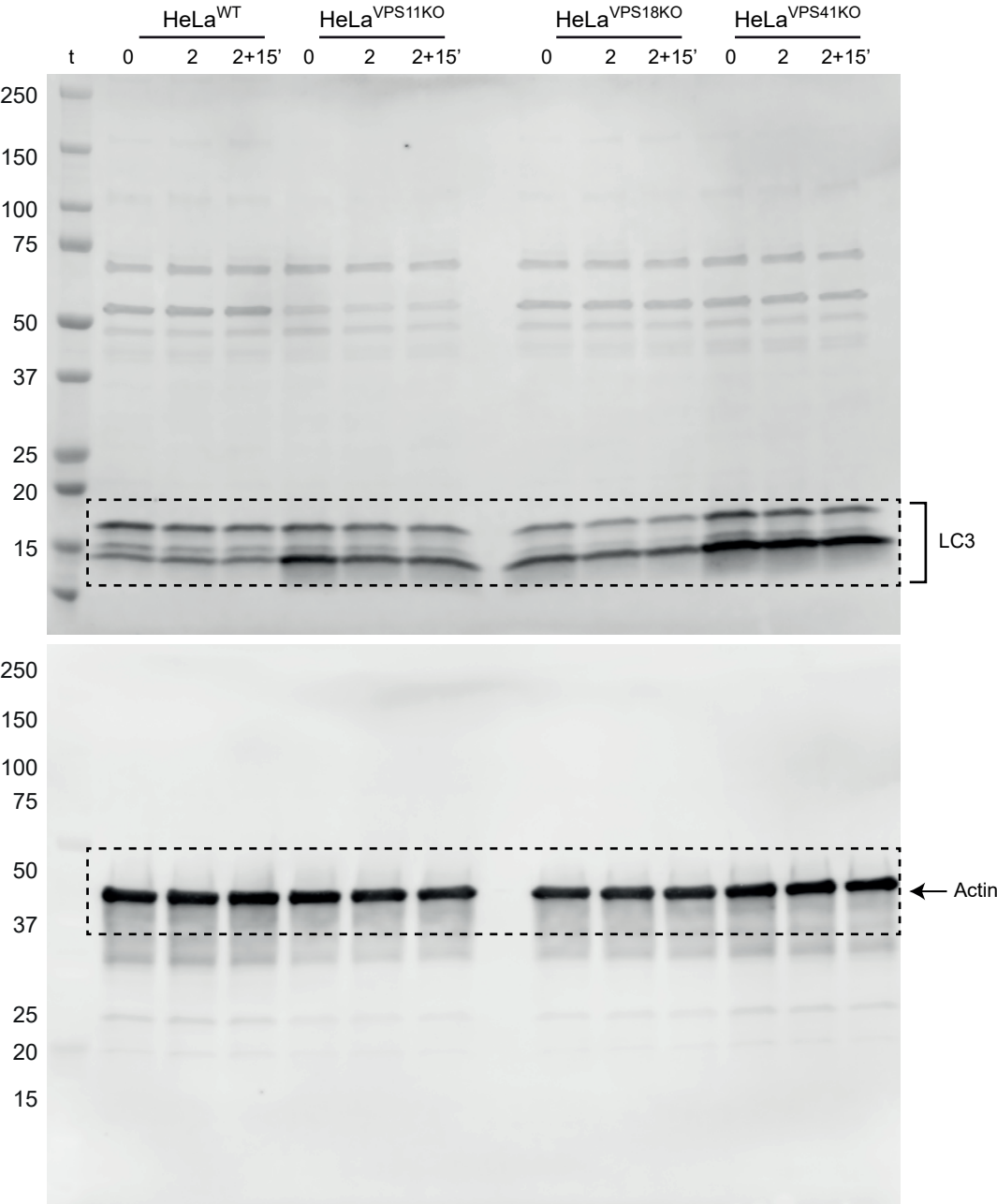

Supplement: Supplementary file 8 — Source Data for Expanded View and Appendix [file EMMM-13-e13258-s013.zip › EV-appendix-SD/EMM-2020-13258-V4_Source_Data_Appendix_Figure_S9.pdf]

Figure 2B

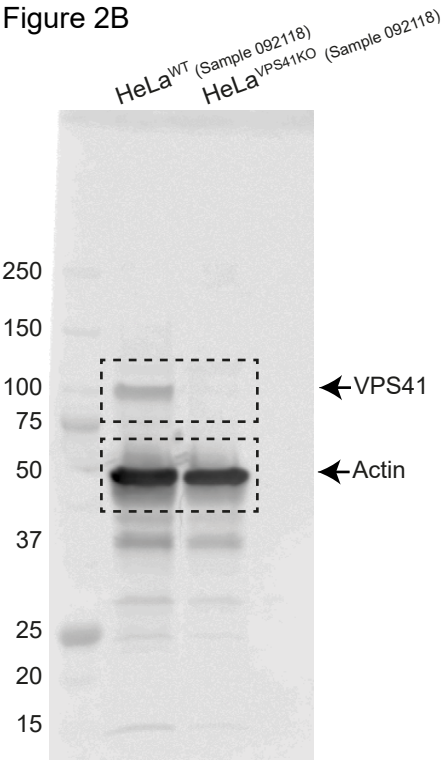

Figure 8C

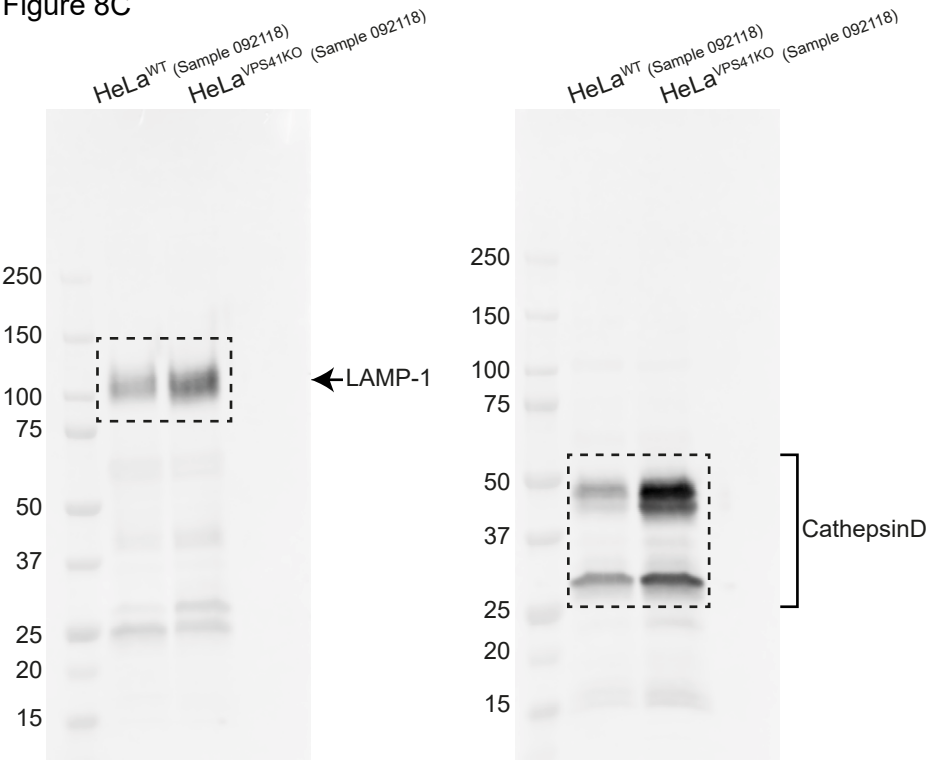

Supplement: Supplementary file 8 — Source Data for Expanded View and Appendix [file EMMM-13-e13258-s013.zip › EV-appendix-SD/EMM-2020-13258-V4_Source_Data_Figure_2_and_Appendix_Figure_S8.pdf]

Figure EV3A

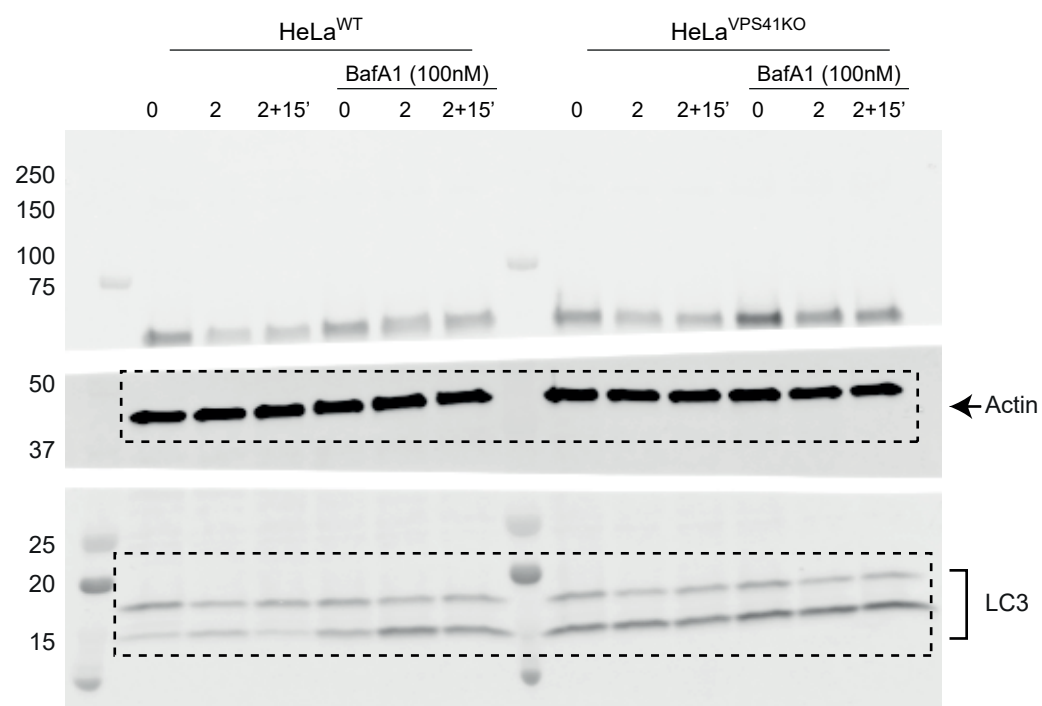

Supplement: Supplementary file 8 — Source Data for Expanded View and Appendix [file EMMM-13-e13258-s013.zip › EV-appendix-SD/EMM-2020-13258-V4_Source_Data_Figure_EV3.pdf]

Figure EV4F

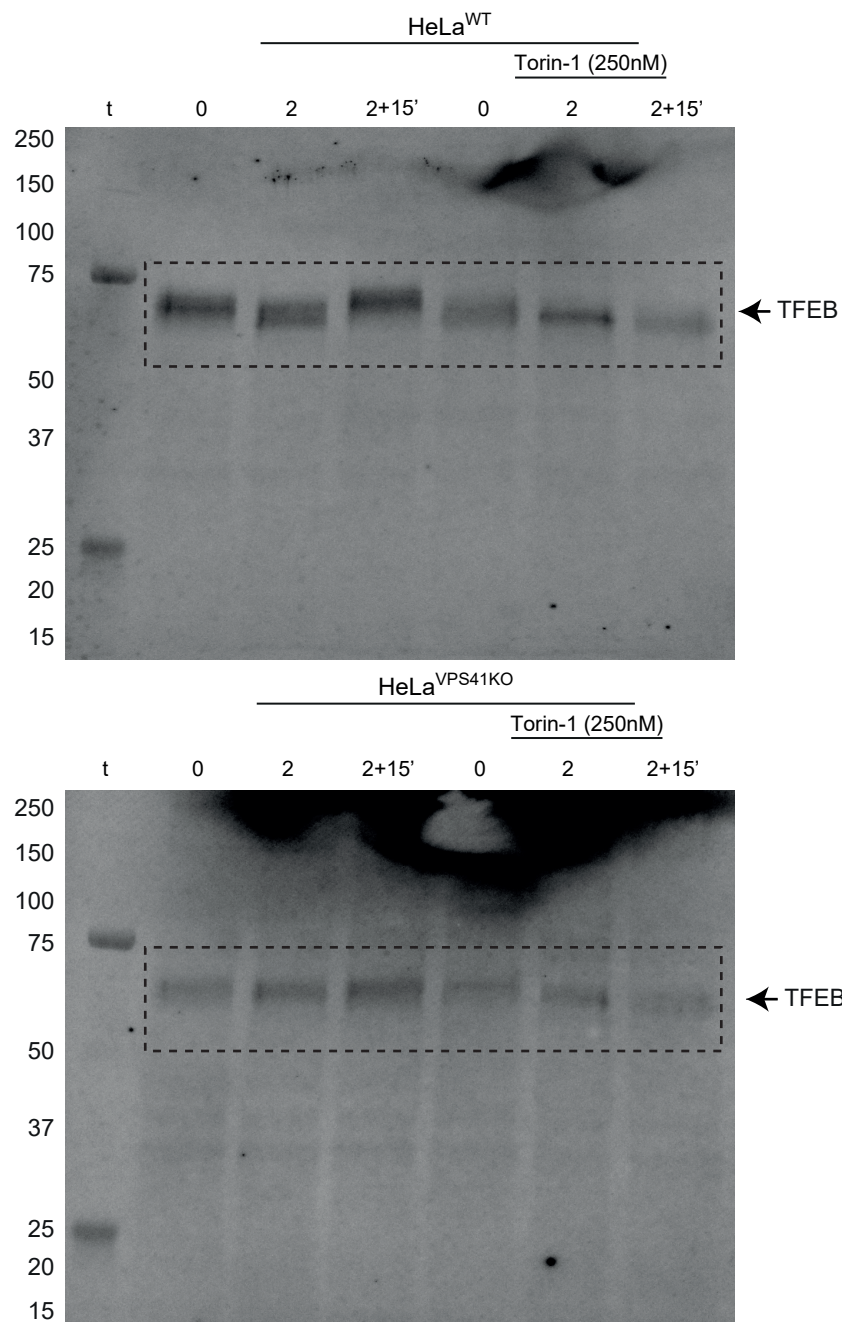

Supplement: Supplementary file 8 — Source Data for Expanded View and Appendix [file EMMM-13-e13258-s013.zip › EV-appendix-SD/EMM-2020-13258-V4_Source_Data_Figure_EV4.pdf]

Figure EV5C

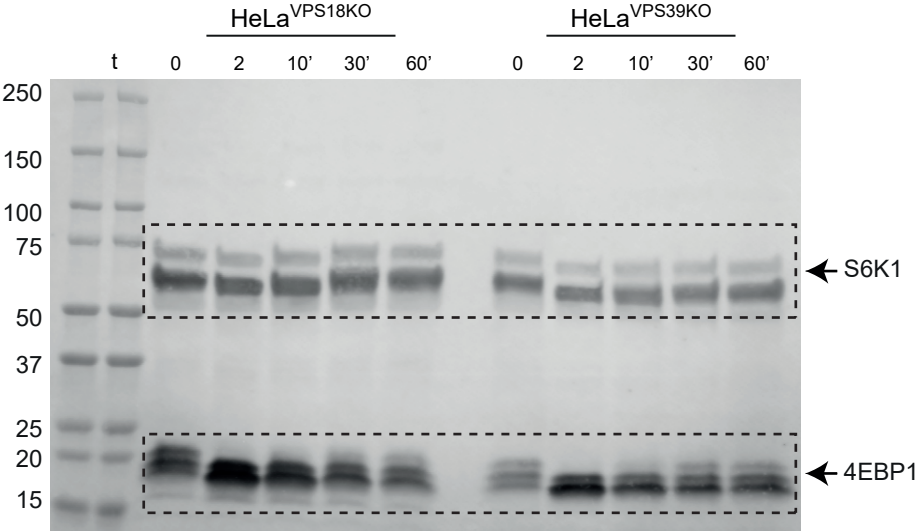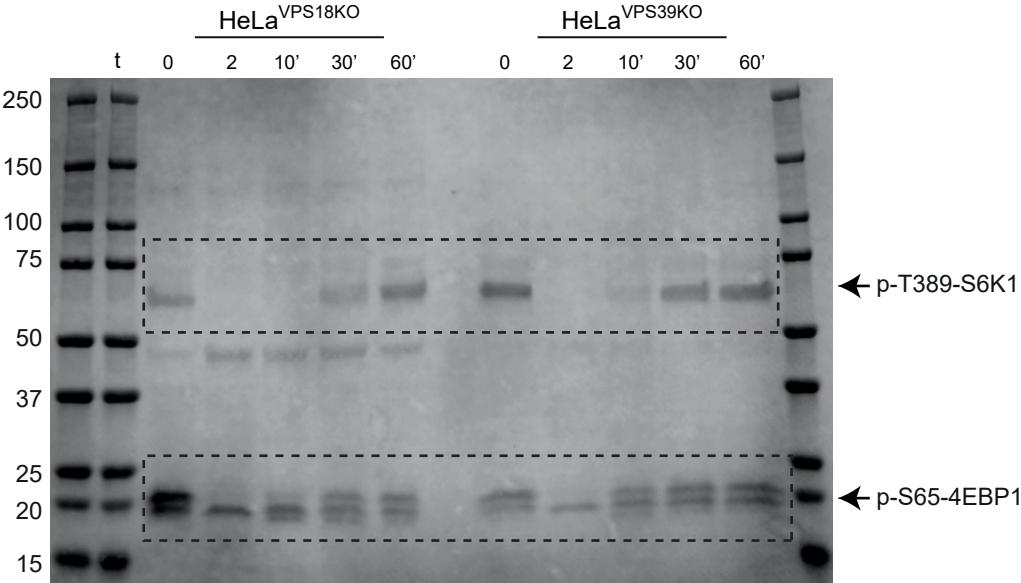

Figure EV5D

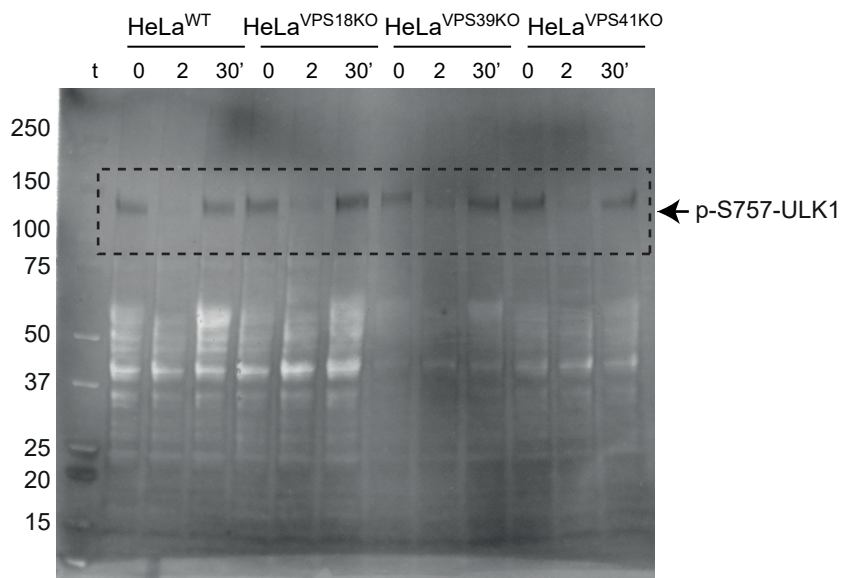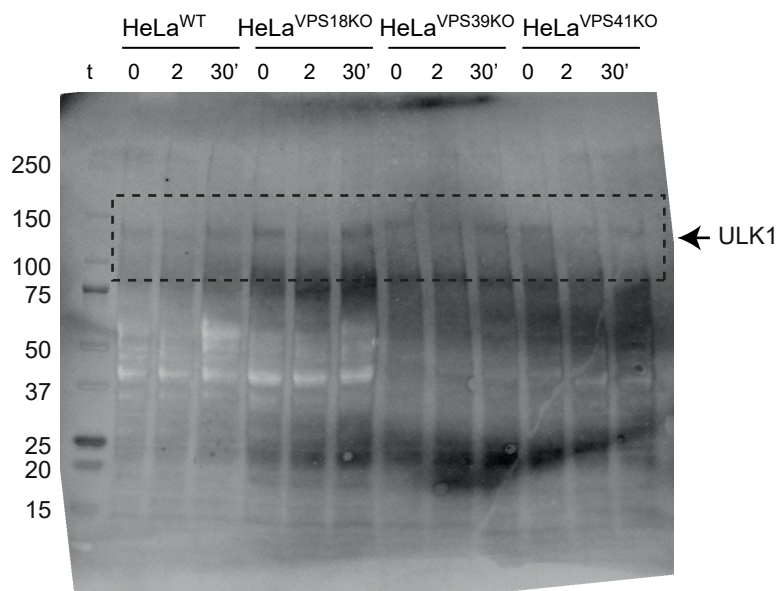

Supplement: Supplementary file 8 — Source Data for Expanded View and Appendix [file EMMM-13-e13258-s013.zip › EV-appendix-SD/EMM-2020-13258-V4_Source_Data_Figure_EV5.pdf]

Figure 3A and Appendix Figure S3A

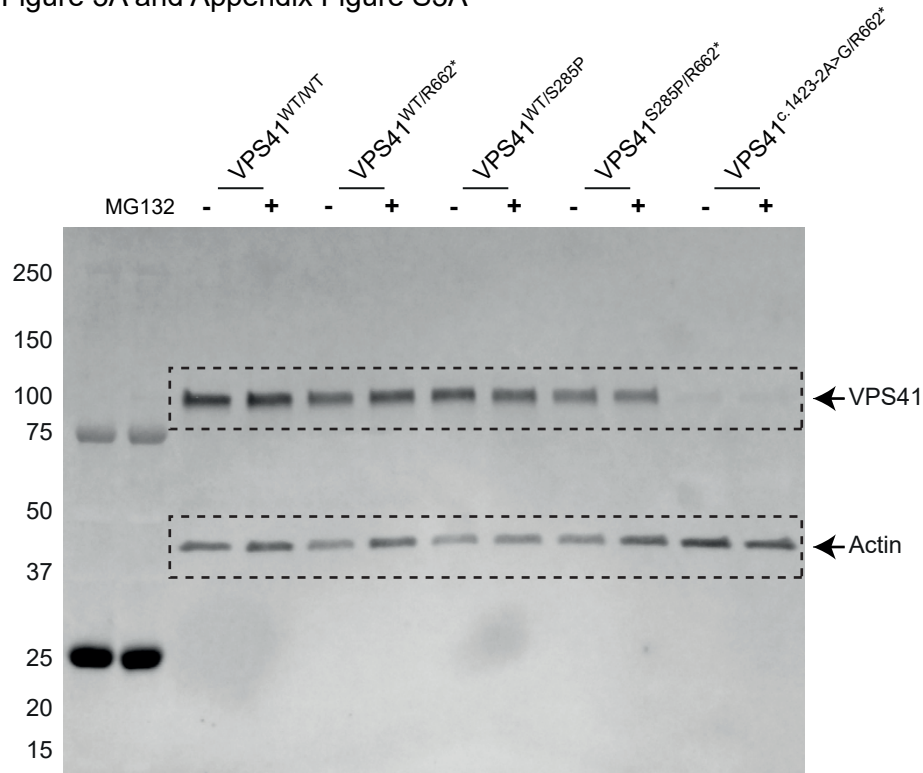

Figure 3B

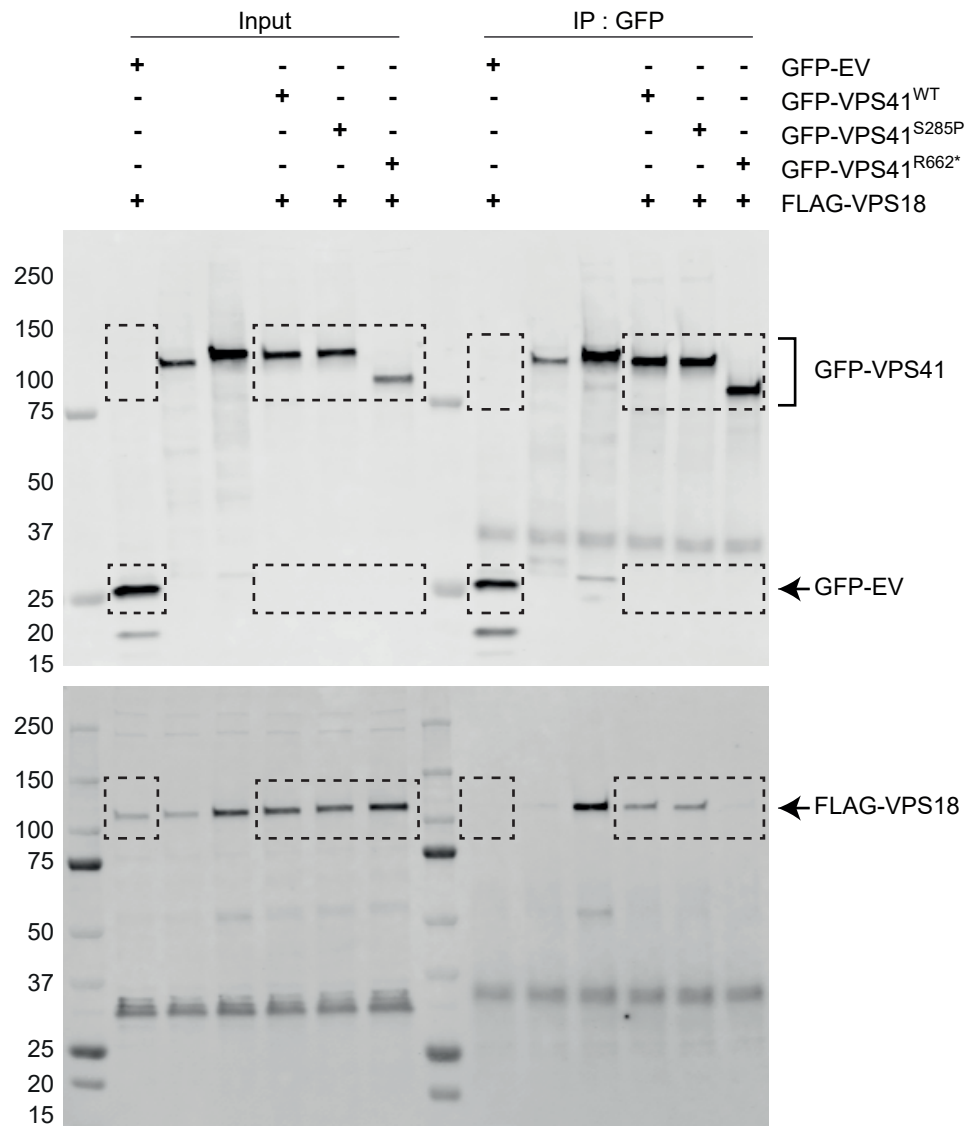

Figure 3C

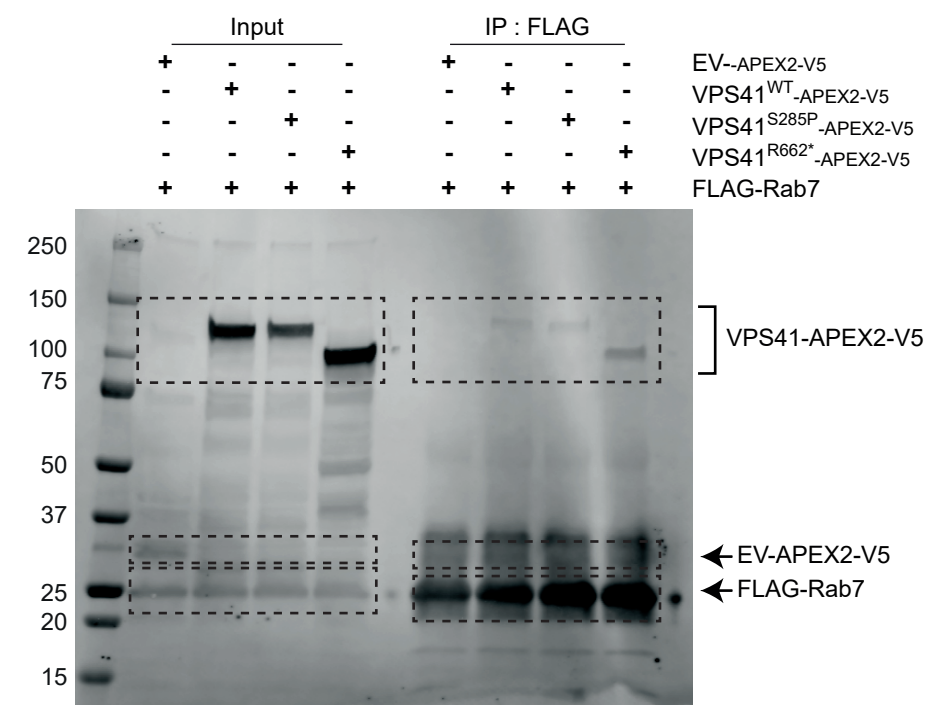

Supplement: Supplementary file 11 — Source Data for Figure 3 [file EMMM-13-e13258-s011.pdf]

Figure 4A

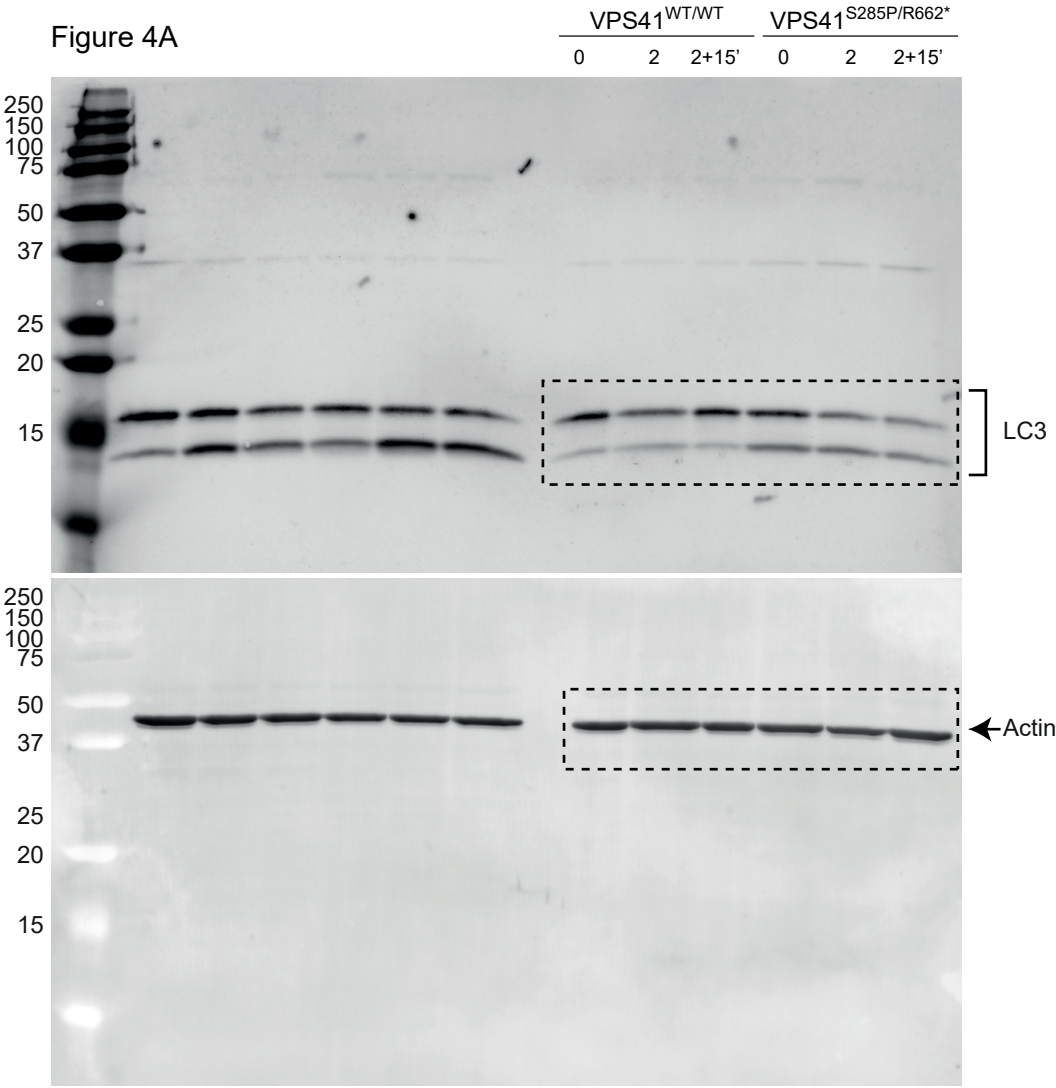

Figure 4C

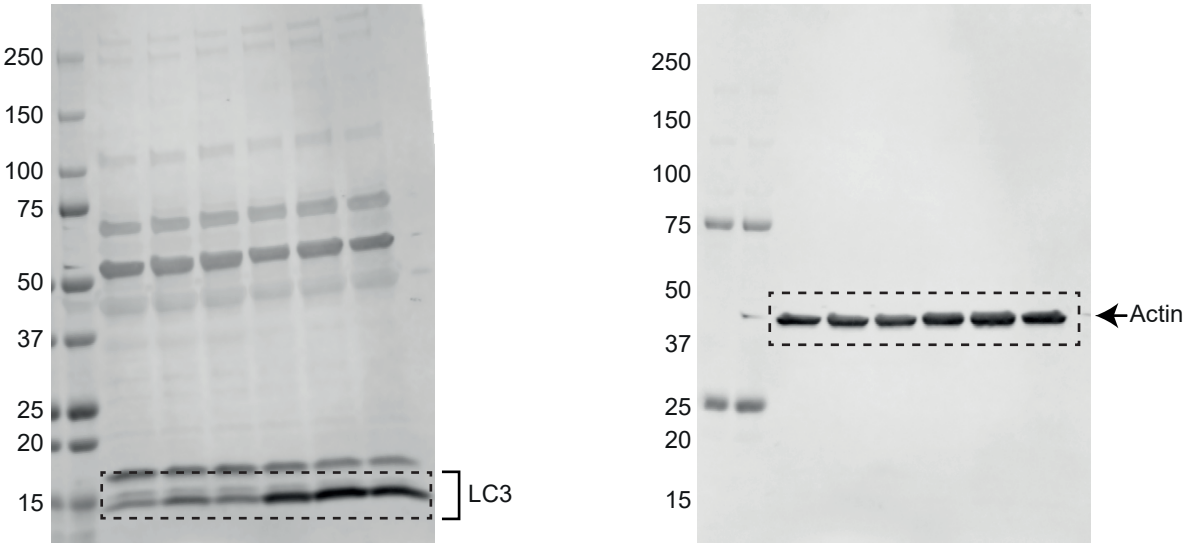

Supplement: Supplementary file 12 — Source Data for Figure 4 [file EMMM-13-e13258-s008.pdf]

Figure 5E

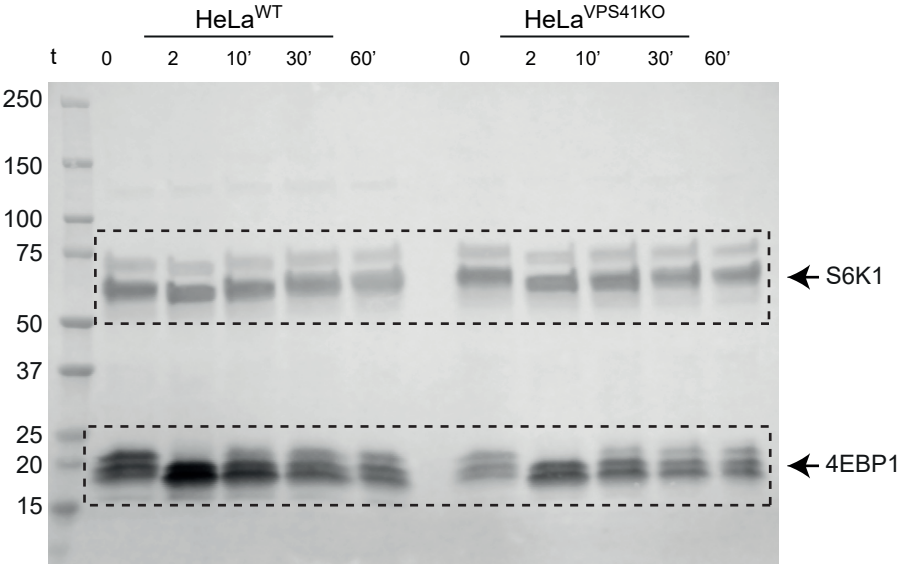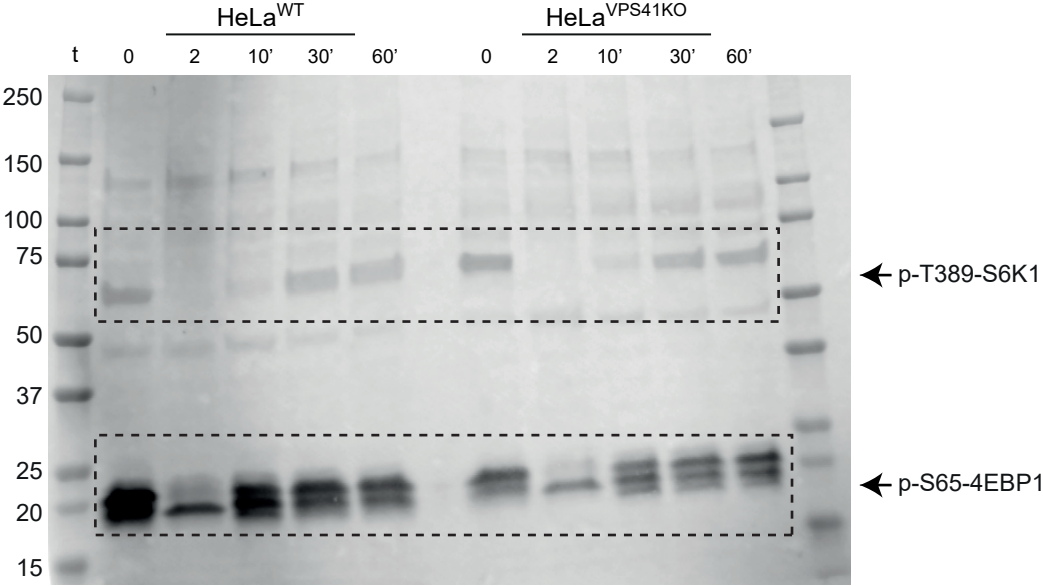

Supplement: Supplementary file 13 — Source Data for Figure 5 [file EMMM-13-e13258-s012.pdf]
